# Gait Characterization and Analysis of Hereditary Amyloidosis Associated with Transthyretin Patients: A Case Series

**DOI:** 10.3390/jcm11143967

**Published:** 2022-07-07

**Authors:** Maria do Carmo Vilas-Boas, Pedro Filipe Pereira Fonseca, Inês Martins Sousa, Márcio Neves Cardoso, João Paulo Silva Cunha, Teresa Coelho

**Affiliations:** 1 Centro Hospitalar Universitário do Porto, Hospital Santo António, Unidade Corino de Andrade, E.P.E., Largo do Prof. Abel Salazar, 4099-001 Porto, Portugal; marciocardoso.neurofisiologia@chporto.min-saude.pt (M.N.C.); tcoelho@netcabo.pt (T.C.); 2 INESC TEC (Instituto de Engenharia de Sistemas e Computadores, Tecnologia e Ciência), FEUP (Faculdade de Engenharia da Universidade do Porto), University of Porto, R. Dr. Roberto Frias, 4200-465 Porto, Portugal; jpcunha@inesctec.pt; 3 LABIOMEP: Porto Biomechanics Laboratory, University of Porto, R. Dr. Plácido de Costa, 91, 4200-450 Porto, Portugal; pedro.labiomep@fade.up.pt (P.F.P.F.); inesmartinsdesousaa@gmail.com (I.M.S.); 4 Escola Superior de Biotecnologia, Universidade Católica Portuguesa Rua de Diogo Botelho, 1327, 4169-005 Porto, Portugal

**Keywords:** ATTRv amyloidosis, clinical neurology, peripheral neuropathy, gait analysis, movement quantification, Familial Amyloid Polyneuropathy

## Abstract

Hereditary amyloidosis associated with transthyretin (ATTRv), is a rare autosomal dominant disease characterized by length-dependent symmetric polyneuropathy that has gait impairment as one of its consequences. The gait pattern of V30M ATTRv amyloidosis patients has been described as similar to that of diabetic neuropathy, associated with steppage, but has never been quantitatively characterized. In this study we aim to characterize the gait pattern of patients with V30M ATTRv amyloidosis, thus providing information for a better understanding and potential for supporting diagnosis and disease progression evaluation. We present a case series in which we conducted two gait analyses, 18 months apart, of five V30M ATTRv amyloidosis patients using a 12-camera, marker based, optical system as well as six force platforms. Linear kinematics, ground reaction forces, and angular kinematics results are analyzed for all patients. All patients, except one, showed a delayed toe-off in the second assessment, as well as excessive pelvic rotation, hip extension and external transverse rotation and knee flexion (in stance and swing phases), along with reduced vertical and mediolateral ground reaction forces. The described gait anomalies are not clinically quantified; thus, gait analysis may contribute to the assessment of possible disease progression along with the clinical evaluation.

## 1. Introduction

Hereditary amyloidosis associated with transthyretin (ATTRv amyloidosis), once known as Familial Amyloid Polyneuropathy, is a rare autosomal dominant disease characterized by polyneuropathy due to amyloid deposition in the peripheral nerves and major organs [1]. More than 120-point mutations related to ATTRv amyloidosis and nerve degeneration have been identified, with the most common cases linked to the replacement of valine by methionine at position 30 of the TTR protein (V30M). This has led to the current designation of this condition as V30M ATTRv amyloidosis.

V30M ATTRv amyloidosis is a highly disabling multisystemic disorder with variable onset and penetration worldwide [2]. The global prevalence has been recently estimated by Schmidt et al. [2] to be around 10,000 persons, although considerable uncertainty exists (range 5526–38,468). In Northern Portugal, where this pathology is endemic, the latest epidemiologic study reported a prevalence of 163.1 per 100,000 adult inhabitants [2]. A prevalence increase of 16% was reported for the Portuguese cities with the highest prevalence (Vila do Conde and Póvoa de Varzim) in the last 21 years [3]. In other countries, the reported prevalence was 104 per 100,000 inhabitants in the northern region of Sweden in 2018 [2]; 1.1–1.55 per 100,000 inhabitants in Nagano (Japan), in 2005 [4]; and 3.72 per 100,000 in Cyprus, in 2003 [5].

The disease presents itself as a nerve length-dependent symmetric polyneuropathy that typically starts at the feet with loss of temperature and pain sensations. It is associated with life-threatening autonomic dysfunction, leading to cachexia and death within 7.3 to 11 years from onset, if left untreated [6]. The natural course of this condition is classified into three stages: I—patients are ambulatory, have mostly mild sensory, motor, and autonomic neuropathy in the lower limbs; II—patients are still ambulatory but require assistance and have mostly moderate impairment progression in the lower limbs, upper limbs, and trunk; and III—patients are bedridden or wheelchair bound and present severe sensory, motor, and autonomic involvement of all limbs [7].

Regarding treatment, liver transplantation has often been the only option for these patients. In recent decades, however, other therapeutic strategies have been developed, such as TTR stabilizers (e.g., tafamidis, indicated for stage I patients, especially for women with slow disease progression [8]), small interfering RNAs (e.g., Patisiran, indicated for stage II, which is intravenous and not indicated for patients with prevalent cardiac involvement [9]) and antisense oligonucleotides (e.g., Inotersen, also indicated for stage II, which affects the kidney and platelets volume [10]). However, liver transplantation is still the most effective and affordable option for V30M ATTRv amyloidosis patients, as management strategies lack cohesion and patients experience years of misdiagnosis and negligible treatment [11].

Motor function of V30M ATTRv amyloidosis patients is currently evaluated with a comprehensive neurological examination, which may include nerve conduction studies with sympathetic skin response (SSR), quantitative sensory testing [12] and self-report questionnaires, such as the Norfolk Quality of Life—Diabetic Neuropathy (QoL-DN) questionnaire [13]. Direct observation followed by a qualitative assessment of movement-associated symptoms based on rating scales is also an approach frequently used [14,15]. The gait pattern of V30M ATTRv amyloidosis patients has been described as similar to that of diabetic neuropathy, associated with steppage gait, loss of dorsiflexion and consequent foot drop and high lifting of the leg [1,16]. On visual inspection, patients spread the legs to improve balance, exaggerating knee and hip flexion and “throwing” the feet forward, as a compensatory strategy in order to improve ground clearance.

There are several different ways of performing gait analysis, with the optic camera-based systems being described as highly accurate [17]. These systems determine a point-position of specific anatomical landmarks on the subject’s body, with a high time and spatial resolution. Multiple infrared cameras can be used to compute a 3D trajectory [18], but other than some markers placed directly to the skin, there are no more constraints to the patient’s movement [18]. Despite the advantages of the quantification of gait characteristics with the use of motion capture technology, this is still relatively rare in neurological conditions [14], and an exploratory subject with patients with V30M ATTRv amyloidosis [19].

The objective of this study is to quantitatively characterize the gait pattern of patients with V30M ATTRv amyloidosis, thus providing information for a better understanding of the loss of function and with potential for supporting diagnosis and progression evaluation. To the best of our knowledge this analysis has not yet been reported with patients suffering of V30M ATTRv amyloidosis, with only one study reporting a selection of spatiotemporal and angular parameters obtained with a RGB-D camera [20] and another using a machine learning model to distinguish between healthy and V30M ATTRv amyloidosis mutation carriers (with or without symptoms), also using gait information recorded with a RGB-D system [21].

Due to the lack of information in the scientific literature [20], this study’s objective is to present an ATTRv V30M amyloidosis patients’ gait quantitative characterization over a period of 18 months. Since this is a rare disease, this study is structured as a case series reporting the gait pattern of five V30M ATTRv amyloidosis patients.

## 2. Materials and Methods

### 2.1. Participants

A group of five patients from the V30M ATTRv amyloidosis unit of the Hospital Santo António—Centro Hospitalar Universitário do Porto (Porto, Portugal) were invited to participate in this study. All the participants had the V30M mutation, although presenting different impairments, such as gait abnormalities, muscular weakness, pain, thermal or tactile anesthesia, or reduced proprioception.

The exclusion criteria were defined and assessed by a neurologist as the presence of orthopaedic, musculoskeletal, rheumatically or cardiovascular constraints that might impair locomotion, and other neurological conditions not associated to the pathology under study. Gait analysis of this group was performed twice: at an initial assessment (T0) and at a second assessment 18 months later (T1). The participants’ demographic and clinical data can be consulted in Table 1.

This study was authorized by the Centro Hospitalar Universitário do Porto Ethics Committee with the protocol number 2014/167(119-DEFI/149-CES), in accordance with the Declaration of Helsinki. All participants read and signed an informed consent form prior to any data collection.

### 2.2. Clinical Assessment

The Medical Research Council Scale (MRC) was applied to the patients by a neurologist in order to assess the state of each analyzed movement: (0) no contraction, (1) flicker or trace of contraction, (2) active movement with gravity eliminated, (3) active movement against gravity, (4) active movement against gravity and resistance, and (5) normal strength [22]. A minus (−) or plus (+) sign was introduced to characterize the movement against a smaller or stronger resistance exerted by a physician, respectively.

Additionally, the Polyneuropathy Disability score (PND) was applied as: (0) no impairment, (I) sensory disturbances in extremities but preserved walking capacity, (II) difficulties in walking but without the need for a walking stick, (IIIa) one stick or one crutch required for walking, (IIIb) two sticks or two crutches required for walking and (IV) patient confined to a wheelchair or bed [23].

The Transthyretin Familial Amyloid Polyneuropathy (TTR-FAP) score was applied as: (Stage 0) asymptomatic; (Stage I) mild, ambulatory, symptoms at lower limbs limited; (Stage II) moderate, further neuropathic deterioration, ambulatory but requires assistance; (Stage III) severe, bedridden/wheelchair bound with generalized weakness.

### 2.3. Experimental Setup

Kinematic data was recorded using a 11-camera Oqus system (Qualisys AB, Gotenburg, Sweden) operating at a sampling frequency of 200 Hz. Prior to each session the camera system was calibrated with a maximum acceptable error of 0.7 mm. Ground reaction forces were collected with five resistive (Bertec, Columbus, OH, USA) and one piezoelectric (Kistler, Winterthur, Switzerland) force platforms, operating at a sampling frequency of 2000 Hz, and in synchrony with the motion capture system. The force platforms occupied an area of 2.4 m by 0.9 m.

**Figure 1:**
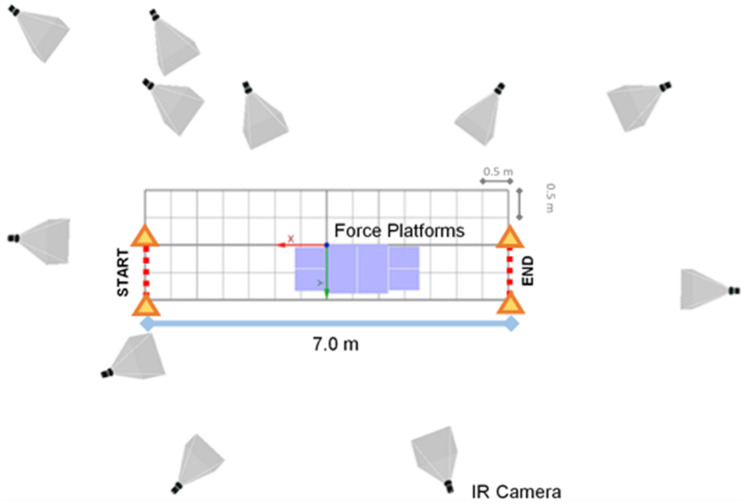
Representation of the gait analysis path with its dimension and limits. The setup of the motion capture cameras is represented by their viewing cones, and the purple squares represent the force platforms.

The gait analysis area was defined as a region of 7.0 m length and 1.0 m width, with the first pair of force platforms placed at its midpoint, and delimited by a pair of signaling cones, as depicted in Figure 1. This region coincided with the motion capture system calibrated volume.

### 2.4. Marker Setup and Biomechanical Model

A lower-limb marker setup was used, comprising thirty-two passive retro-reflective markers placed over relevant anatomical landmarks. Markers were placed on the right and left anterior and posterior iliac spines, at the right and left trochanter, on the right and left lateral and medial femur epicondyles, on the right and left tibial tuberosity, on the right and left head of the fibula, on the right and left lateral prominence of the lateral and medial malleolus, on the right and left distal end of the posterior aspect of the calcaneus, on the right and left lateral aspect of the first and fifth metatarsal head, and on the dorsal aspect of the second metatarsal head. Additionally, four-marker clusters were positioned on right and left thighs and shanks, according to the CAST marker set [24,25].

### 2.5. Experimental Procedures

Participants were instructed to walk naturally and barefoot at a comfortable self-selected pace, back and forth, along the analysis path. At least 10 valid trials were performed by each participant.

Patients who normally used walking aids or splints did not use them for this experiment. Additionally, a research assistant accompanied the participant along the path and was prepared to help in case of difficulties during the task.

### 2.6. Data Processing

After data collection, the Qualisys Track Manager (Qualisys AB, Gotenburg, Sweden) software was used to review and identify each marker trajectory, and trajectory gaps were interpolated using the built-in polynomial calculations. The resulting processed data was then exported to the Visual3D software (C-Motion, Inc., Germantown, MD, USA) for further processing and analysis, including trajectory filtration with a 6 Hz bidirectional low-pass Butterworth filter and the creation of a six degrees of freedom anatomical model. A global and local coordinate system (for each segment) has been defined in which the X axis corresponds to the lateral (+) and medial (−) directions, the Y axis corresponds to the anterior (+) and posterior (−) directions, and the Z axis corresponds to the cephalic (+) and caudal (−) directions [26]. Gait events were calculated automatically with the appropriate Visual3D built-in routine, and included heel strike (HS), midstance (MS) and toe off (TO). Joint angles were calculated using the rotation order of the distal segment with respect to the proximal segment, applying each segment’s local coordinate system [26]. Lower-limb angles were assigned with three rotational degrees of freedom and calculated using an XYZ Cardan sequence of rotations, which are equivalent to flexion/extension, abduction/adduction and axial rotation, respectively. Hip flexion, knee flexion, and ankle dorsiflexion were displayed as positive angular displacement.

Linear and angular kinematics, as well as the corresponding ground reaction forces were retrieved. Linear kinematics included gait speed, stride length and width, step length, cadence (steps/minute), as well as gait cycle, stance, swing and double limb support duration. Angular metrics were extracted at the instant of left and right heel strike (HS), midstance (MD) and toe-off (TO).

Angular kinematics were time-normalized to the gait cycle (heel strike to heel strike), while ground reaction forces were normalized to the stance duration (heel strike to toe off). Ground reaction forces were also amplitude-normalized and expressed as a percentage of the participant’s body weight (BW). Events were calculated for the characterizing points in the anterior-posterior (FAP), medial-lateral (FML) and vertical (FV) force vectors, and numbered consecutively, according to [25].

The reference gait data for non-pathological individuals used as comparison in this study were retrieved from the Qualisys Clinical Gait Plug-In analysis module.

### 2.7. Statistical Analysis

Data normality was assessed using the Kolmogorov–Smirnov test for variables over 50 data points, or the Shapiro–Wilk test when less than 50 data points were available. For parametric data, a paired sample *t*-test and effect size calculation was performed between T0 and T1. Effect size was evaluated according to the $\eta_{\varrho }{}^{2}$ value [27]. Results were interpreted as small (0.01), moderate (0.06) or large (0.14) [27]. For non-parametric data, the Wilcoxon signed rank test was performed and effect size calculated as *Cohen’s d*, and interpreted as small (0.1), moderate (0.3) or large (0.5) [27]. Descriptive statistics were computed for each subject and are presented as mean (standard deviation) or median [interquartile range] for parametric and non-parametric variables, respectively.

All statistical procedures were performed using SPSS 26 (IBM, New York, NY, USA) and a significance level of α = 0.05 was used.

## 3. Results

### 3.1. Clinical Assessment

The results from the clinical assessment of each participant revealed different scores, indicating distinct progression and manifestation of the pathology. These results are presented in Table 2.

They also presented some degree of motor deficit at the lower limbs: one patient had no strength deficit but had slight difficulty in walking on heels (P3), while the others had ankle dorsiflexion strength from 4/5 to 0/5, in the MRC scale [22], and ankle plantar-flexion from 4/5 to 1/5. All patients had normal knee segmental force (5/5), except P2 which had knee flexion and extension 4/5. All patients had flexion/extension of the toes between 0 and 3, and absent Achilles reflexes in both T0 and T1. Overall, patients presented sensory ataxia and steppage gait with different instability and movement coordination degrees during stride. They presented heterogeneous gait, although the clinical perception is that all alterations resulted from the sensory-motor polyneuropathy caused by the disease.

### 3.2. Summary of Results

Complete results are shown in Appendix A: Linear Kinematics, Appendix B: Ground Reaction Forces, and Appendix C: Angular Kinematics.

#### 3.2.1. Linear Kinematics

A significant decrease in gait speed (P1: −13.51%, P2: −16.53%, P5: −5.62%) was observed at T1, associated with a shortening of the step length (but not stride width or length) and a lower cadence. These alterations also affected the gait cycle duration, which increased, along with the stance phase duration. In general, V30M ATTRv amyloidosis patients show longer gait cycles, associated with longer double limb support and shorter steps. There is no significant increase in step width, as one would expect, at least for P4 which went from “very low steppage”, at T0 to formal “steppage” at T1. The patient P3 shows the least changes between sessions.

#### 3.2.2. Ground Reaction Forces

**Figure 2:**
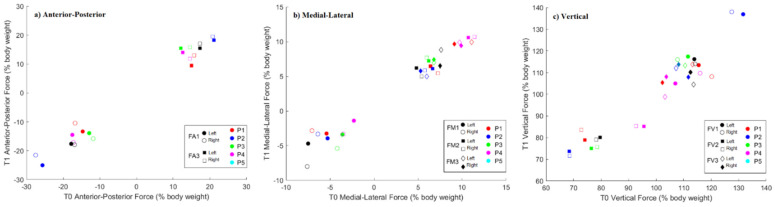
Scatterplot of the ground reaction force (% body weight) produced by each subject and limb at T1 in respect to T0 in the (**a**) anterior-posterior, (**b**) medial-lateral and (**c**) vertical directions, at the respective force characterizing events. FA1: maximum posterior loading force; FA3: maximum anterior thrusting force; FM1: first maximum lateral force; FM3: second maximum medial force; FV1: first maximum vertical loading force; FV2: dip trough force; FV3: second maximum vertical thrusting force.

The ground reaction forces recorded at T0 and T1 for the left (LLL) and right (RLL) lower limbs were recorded and compared. Figure 2 shows a representation of the (a) anterior-posterior, (b) medial-lateral and (c) vertical ground reaction forces produced by each subject at T1 as a function of the values recorded at T0. A full description of the ground reaction values can be consulted in Appendix B.

Between evaluation sessions, significant alterations occurred in the ground reaction forces generated during gait, which were more expressive in the RLL (P1 and P4). Significant differences with moderate to large effect size were found for both limbs at maximum anterior thrusting force (FA3) for both patients, at the first maximum lateral force (FM1) for P1, and at the dip trough and second maximum vertical forces, FV2 and FV3, respectively, for P4. P5 showed significant alterations (with large effect size) in the FA3 and FM1 ground reaction forces generated during gait. From Figure 2 it is possible to see that FM1, FM3 and also FV3 are the force peaks that, in general, show the most prominent changes from T0 to T1.

P2 and P3 did not show significant alterations in the ground reaction forces generated during gait between T0 and T1. For P2, effect size was generally higher for RLL and at FV1 and FM1 for both limbs, and the patient shows lower FA1/FV2 and higher FA3/FV1. For P3, only the FV2 of the left limbs showed high effect size.

Regarding the healthy values described in the literature [28], P1, along with P3 and P4, show a lower maximum posterior loading force (FA1) than the mean reference value (20% body weight). P3 and P4 have a lower first maximum medial force (FM1, between 5 and 10% body weight). With regards to the first maximum vertical force (FV1) P1 shows a lowering in RLL cycles at T0, P2 shows a higher peak and all other patients show a lower value than normal (around 120% body weight). Minimum vertical force (FV2) is higher than normal (around 70% body weight) for P1, at T1, and for both lower limbs cycles for P3, P4 and P5. The maximum vertical force (FV3) is lower than usual (around 120% body weight) for all patients.

#### 3.2.3. Angular Kinematics

A detailed description of each participant’s joint angles at the analyzed gait events, as well as a representation of the joint angles during the gait cycle can be consulted in Appendix C.

In the three analyzed planes (sagittal, frontal and transverse), P1 showed a statistically significant difference with a large effect size in heel strike (HS) from T0 to T1 (27 of the 42 registered instances, 15 in the left and 12 in the right lower limb). Midstance (MD) and toe-off (TO) showed statistically significant differences in 20 and 22 moments, respectively (12 left and 8 and 10 right, each phase). P2 showed a statistically significant difference with a large effect size from T0 to T1 for 30 of the 42 HS analyzed (18 left and 12 right), 28 MD (14 left and 14 right) and 30 TO (16 left and 14 right). For P3, 22 HS (10 left and 12 right), 19 MD (11 left and 8 right) and 17 TO (8 left and 9 right) presented statistically significant differences from T0 to T1, along with a large effect size. P4 showed the following differences between assessments with 25 HS (12 left and 13 right), 22 MD (10 left and 12 right), and 21 TO (12 left and 9 right). P5 has the higher amount of differences between assessments 35 HS (16 left and 19 right), 35 MD (17 left and 18 right), and 37 TO (18 left and 19 right).

With regards to angular kinematics, graphical representation of the pelvis, hip, knees and ankles is presented in Appendix C. Statistical difference was explored for the different moments (T0 and T1), limbs (right and left) and between patients and reference gait data mean values for the three planes sagittal, frontal and transverse.

In summary, the results show that there is a general tendency to delay the toe-off at T1 (P1, P2, P3 and P5). All patients show a more retroverted pelvis than the reference data. P1, P2, and P4 show more prominent left and right pelvic rotation at T1 than T0. P1, P2, P3 and P5 show higher hip extension than the reference gait data. Excessive transverse rotation is also observed for the same participants. For all patients the knee flexion of the stance and swing phases (before and after the toe-off mark) is higher at T1 than T0 and also than the reference data. For P1, P2, P3 and P4, the transverse plane shows a tendency for the right lower limb cycles to show a more external rotation of the knee and the left lower limb cycles a more internal rotation. The ankle angle shows a higher dorsiflexion than the reference gait data before the toe-off for all participants, as well as higher ankle plantar-flexion immediately after the initial contact. In the transverse plane, P1, P2 and P3 show a generally more prominent internal rotation of the ankle than in the reference gait data.

## 4. Discussion

The goal of this study was to quantitatively characterize the gait pattern of patients with V30M ATTRv amyloidosis, thus providing information for a better understanding and potential for supporting disease progression. Laboratory gait analysis is an important part of the clinical evaluation of patients with complex locomotor disability and is claimed to improve the clinical outcomes [17].

We assessed five V30M ATTRv amyloidosis patients twice (18 months apart), using a 12-camera, marker-based, optical system as well as six force platforms. Linear kinematics, ground reaction forces, and angular kinematics results are analyzed for all patients. All patients, except one, showed a delayed toe-off in the second assessment, as well as excessive pelvic rotation, hip extension and external transverse rotation and knee flexion (in stance and swing phases), along with reduced vertical and medial-lateral ground reaction forces.

Our findings reveal that in general, V30M ATTRv amyloidosis patients show longer gait cycles, associated to longer double limb support time and shorter steps. In diabetic neuropathy, these alterations have been associated with decreased muscle strength of the ankle dorsiflexors and plantar-flexors [29]. All except one patient showed a delayed toe-off between assessments, increasing the stance phase and overall cycle time. All patients show a more retroverted pelvis than the reference data. Pelvic rotation was higher than for the reference healthy population for the majority of the patients, some even with a higher angular variation which denotes pelvic instability. On the contrary, P3 who is the patient with the minimal clinical abnormalities, shows a minimal pelvic rotation (around 1 degree, in the transverse plane) at T1. The normal rotation of about 4 degrees on either side of the central axis has the effect of smoothing the vertical dislocation of the center of mass and reducing the impact at foot strike [28], which may be difficult for the referred patient.

Our results show that the hip flexion-extension active range of motion, in the sagittal plane, is more prominent in ATTRv V30M amyloidosis patients (50 to 60 degrees) than in the reference population (around 45 degrees). Adduction and transverse external rotation are also more present in the pathological group, in different gait cycle phases, than in the reference data. Abnormal hip rotation may result from a compensatory movement. External rotation, in particular, may be used to facilitate hip flexion, using adductors as flexors [17].

Regarding the stance phase knee flexion, it is shown to be higher for patients than for the reference gait data and increases from one assessment (T0) to the other (T1). The same happens to the swing phase knee flexion. Excessive knee flexion usually follows abnormal initial contact, occurring to compensate for excessive plantar-flexion, without which the foot would drag. For a diabetic neuropathy group described in the literature, compared with reference group values, the maximum knee joint angle was smaller, in the sagittal plane [30]. A significantly reduced level of peak torques at the ankle and knee in a diabetic polyneuropathy group was also reported [31]. Steppage, which is present in P2, P3, P4 and P5, is a swing phase alteration consisting of exaggerated knee and hip flexion, to lift the foot higher than usual, for increased ground clearance. Usually patients present steppage to compensate for an excessive plantar-flexion—“foot drop”—due to inadequate dorsiflexion control.

All patients in this study show higher ankle plantar-flexion than the reference data immediately after the initial contact. This excessive ankle plantar-flexion during stance has a primary functional penalty which is loss of progression and leads to the shortening of the stride length and reduced gait speed. It also affects stability through the difficulty in maintaining the upright posture. It may be caused by weakness of the pretibial muscles (e.g., the tibialis anterior) which fail to produce an adequate dorsiflexion, allowing the foot to fall in an uncontrolled manner and therefore possibly hampering shock absorption [32]. In this study, patients also presented a higher dorsiflexion during midstance. This may be caused by prolonged heel contact and weakness or impaired control of the soleus which fails to stabilize the tibia, causing a sustained knee flexion. Without a stable foot base, the quadriceps are not able to extend the knee. A higher dorsiflexion may be caused by lack of feet stabilization. Concomitantly, knee flexion may besustained to lower the center of gravity and increase stabilization, or due to the lack of subclinical strength in knee extension (except in the case of P2 that has MRC 4 for knee strength). P3, although exhibiting a close to normal neurological examination, shows an interesting almost permanent dorsiflexion, which has been described as being associated with an inefficient push-off [28]. Excessive dorsiflexion at the time the heel contacts the floor is rare, and translates a position of instability [32]. The correct foot placement in stance and the adequate clearance of the ground in the swing phase are important requisites for safe walking.

With the GRF analysis, we find that FM1, FM3 and also FV3 are the force peaks that, in general, show the most prominent changes from T0 to T1. With regard to the FMs, these are the most variable of the three components of force and can be easily affected. The second vertical force peak (FV3), in which all patients had a performance lower than the values in the literature [28], relates to the amount of vertical propulsive force, which drives the person upwards. A low peak is associated with a poor ability to push off. Causes for insufficient push off may be associated, among other factors, with the triceps suræ weakness, that in these series was observed in P2, P4 and P5, or pain under the forefoot. Patients with V30M ATTRv amyloidosis may experience foot pain throughout the natural history of the disease [23]. The first peak of vertical force relates to the amount of loading the person is putting onto the front foot. In patients with diabetic neuropathy, the maximum values of the vertical component of GRF were found to be lower than in two control groups [30].

Reduced FA3 (P1, P3, P4 and P5) also shows that the person is not propelling the body forward efficiently. The maximum value of the anteroposterior forces was also found to be higher in a control group than in a diabetic neuropathy group [30]. A reduced loading, as was the observed with most of the ATTRv V30M amyloidosis patients, could relate to the presence of pain, discomfort, poor functional movement of lower limb joints or slow walking speed. Karmakar et al. [33], reported, regarding neuropathic pain, that it influenced gait stability and its potential relief using pharmacotherapy did not improve gait dysfunction.

With regard to disease modifying treatments, P1, P4 and P5 underwent orthotopic liver transplantation (LT), 6, 12 and 18 years before, and they presented 9, 18 and 13 years of disease progression. LT removes the main source of the circulating mutated TTR (over 90%), reduces the rate of axonal degeneration, and was the first available treatment of V30M ATTRv amyloidosis. It is an invasive surgical procedure, with long-term risks and morbidity. Nevertheless, early LT is reported to improve the course of the neuropathy [34,35] and to slow disease progression relative to the natural history of this disease [36]. After the first few years following LT, patients they are considered to be in a phase of almost no progression of the neuropathy due to a reduction of nerve loss in transplanted patients [34]. Clinically, the observed patients have a similar profile at T0 and T1 with only a small worsening (see Table 2), which may be attributed to slight clinical subjective impressions between the two clinical consultations. Furthermore, patients may complain of limb weakness, extreme fatigue, postural hypotension and cardiac involvement that are not generally protected/treated by LT [34]. These too can contribute to some gait changes, which may justify several statistically significant differences between both gait assessments, in the patients of this series. More studies are needed to understand the impact of these variables on gait abnormalities.

P2 and P3 have been taking tafamidis, a TTR stabilizer, for 3.5 years and 1 year, with 8 and 5 years of disease progression, respectively. Tafamidis has been reported as having a protective effect of a few years on those who take the medication from the beginning of the disease onset in contrast to those who started it later [37]. P3 started treatment at year 4 of disease progression and P2 at year 4.5 years. There was practically no clinical evolution between T0 and T1 for either P2 or P3: P2 is in a more advanced moment of disease progression with clear steppage, and P3 showed only a mild sensitive neuropathy, with vibration anesthesia on the hallux and minor difficulties on heels or tip toes gait, in both clinical observations. Nevertheless, they exhibited some of the same gait alterations as the other patients between gait assessments, such as the delayed toe-off, hip extension and knee flexion, which may suggest a slight worsening of the clinical condition, not detected on the clinical evaluation, despite the treatment with tafamidis. Non-responders to this medication have been described in the literature [38].

This study shows gait abnormalities that vary in time and that, nowadays, are not clinically quantified. Gait analysis is an important complement to the clinical assessment to the extent that it shows the overall effects that disease progression is having in daily life. Therefore, this assessment may contribute to and complement the current clinical analysis.

Since this is a rare disease, and our sample includes only a small number of patients, we structured this study as a case series, which seemed to be more useful. Although a case series is frequently incomplete and biased, it may enlighten future study strategies [39], and avoid the effects of data heterogeneity. Group analysis has been described as possibly having a negative impact on understanding the pathophysiology and management of rare diseases, since it may not reflect exactly what happens in individual patients [39]. Nevertheless, individual measurements may not always correspond to average reference values not only because of the disease but also because of the normal variability between individuals.

Adding to the small number of participants, this study has some other limitations including a longer, and single time between assessments, and the heterogenicity of the participants’ clinical condition/disease progression/treatment. Nevertheless, a valuable insight into the problems related to V30M ATTRv amyloidosis characteristic gait pattern has been obtained.

Although V30M ATTRv amyloidosis is a degenerative disease, and patients suffer from muscle weakness, neuralgic pain and sensory loss, all of which contribute to settling into a pathological gait pattern, clinical importance should be given to rehabilitation and maintenance of the functionality of the ankle complex, in order to maintain greater mobility and muscle strength of the ankle for a better gait performance, as suggested for diabetic neuropathy [29]. Further studies are needed, for a more comprehensive assessment of motor control impairment during gait, such as electromyography studies, orthopedic assessment, especially articular, which may also be affected in these patients (e.g., Charcot joint neuroarthropathy). It would also be interesting to specifically design a comparison study with ATTRv amyloidosis and other neuropathies, including the diabetic neuropathy, in order to understand if the different diseases present distinct pathological gait characteristics.

## Figures and Tables

**Table 1:** Demographics and clinical data for each patient that participated in the experiment. All data reports to the time of the first gait analysis, while BMI variation represents the change between analysis periods.

Patient	Gender ^1^	Height (m)	Weight (kg)	Age (Years)	BMI (kg/m^2^)	BMI Variation (kg/m^2^)	Years of Disease Progression	Years since Diagnosis
P1	M	1.72	72.0	34	24.34	0.0	9	8
P2	M	1.73	58.5	33	19.55	1.5	8	8
P3	F	1.68	63.8	48	22.60	0.6	5	2
P4	F	1.48	61.5	54	28.08	−1.4	18	17
P5	M	1.71	53.0	52	18.13	0.9	13	13

^1^ Gender is expressed as male (M) and female (F). BMI stands for body mass index.

**Table 2:** Clinical evolution of the ATTRv V30M patients based on the Medical Research Council (MRC) Scale. The Polyneuropathy disability (PND) and TTR-FAP scores are also indicated.

Patient	MRC ^1^ Scores at T0	MRC ^1^ Scores at T1	PND Score ^2,^*	TTR-FAP Score ^2,^*	Treatment at T0
P1	Dorsiflexion deficit (4), minor vibration anesthesia on the hallux	Dorsiflexion deficit (4−), minor vibration anesthesia on the hallux	II	I	Transplant 6 years ago
P2	Dorsiflexion (0), plantar-flexion (1), knee flexion and extension (4), sensory ataxia and high steppage	Dorsiflexion (0), plantar-flexion (0), knee flexion and extension (4), sensory ataxia and high steppage	II	I	Tafamidis for 3.5 years
P3	Only vibration anesthesia on the hallux, minor difficulties on heels or tip toes gait	Only vibration anesthesia on the hallux, minor difficulties on heels or tip toes gait	II	I	Tafamidis for 1 year
P4	Dorsiflexion (4), plantar-flexion (4), sensory ataxia, low steppage	Dorsiflexion (3), plantar-flexion (4), sensory ataxia, steppage	II	I	Transplant 12 years ago
P5	Dorsiflexion and plantar-flexion (2), sensory ataxia and high steppage	Dorsiflexion (0), plantar-flexion (1), sensory ataxia and high steppage	II	I	Transplant 18 years ago

^1^ MRC scores range from 0 (worst result) to 5 (best result). ^2^ PND and TTR-FAP range from I (*sensory disturbances in extremities but preserved walking capacity and mild, ambulatory, symptoms at lower limbs limited*) to IV (*patient confined to a wheelchair or bed*) or III (*severe, bedridden/wheelchair bound with generalized weakness*), respectively. * PND and TTR-FAP scores were the same in both evaluation periods.

**Figure A1:**
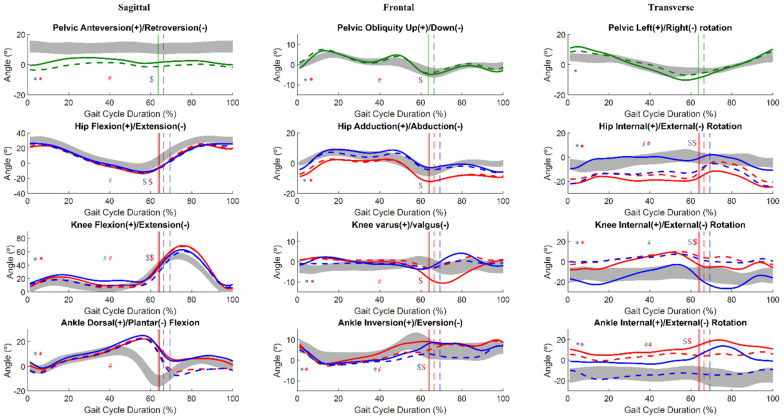
Average pelvis and lower limbs joint angles (in degrees) in the three anatomical planes during a gait cycle of the right (blue), left (red) lower limb and irrespective of the side (green). The joint angles at T0 are denoted by a solid line, while T1 is represented by a dashed line. The reference interval from the healthy gait dataset is shaded grey. A set of symbols are used to denote statistically significant differences, associated with large effect size, between T0 and T1 at the heel strike (*), midstance (#) and toe-off ($), with their color indicating the limb presenting such differences.

**Figure A2:**
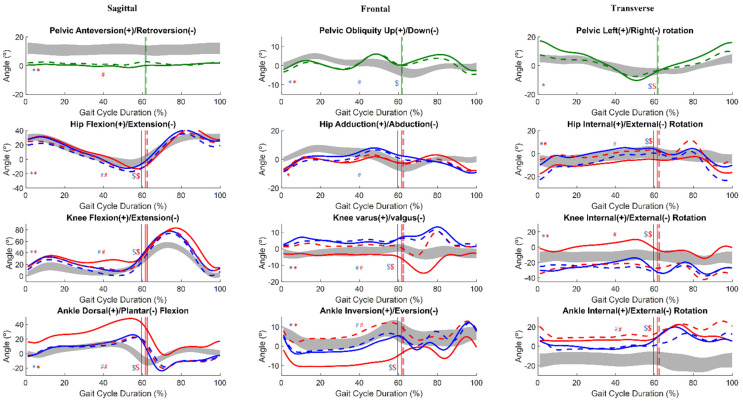
Average pelvis and lower limbs joint angles (in degrees) in the three anatomical planes during a gait cycle of the right (blue), left (red) lower limb and irrespective of the side (green). The joint angles at T0 are denoted by a solid line, while T1 is represented by a dashed line. The reference interval from the healthy gait dataset is shaded grey. A set of symbols are used to denote statistically significant differences, associated with large effect size, between T0 and T1 at the heel strike (*), midstance (#) and toe-off ($), with their color indicating the limb presenting such differences.

**Figure A3:**
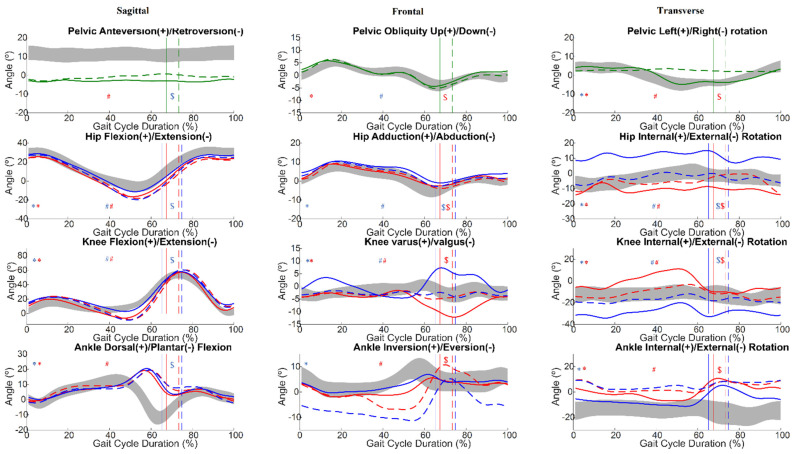
Average pelvis and lower limbs joint angles (in degrees) in the three anatomical planes during a gait cycle of the right (blue), left (red) lower limb and irrespective of the side (green). The joint angles at T0 are denoted by a solid line, while T1 is represented by a dashed line. The reference interval from the healthy gait dataset is shaded grey. A set of symbols are used to denote statistically significant differences, associated with large effect size, between T0 and T1 at the heel strike (*), midstance (#) and toe-off ($), with their color indicating the limb presenting such differences.

**Figure A4:**
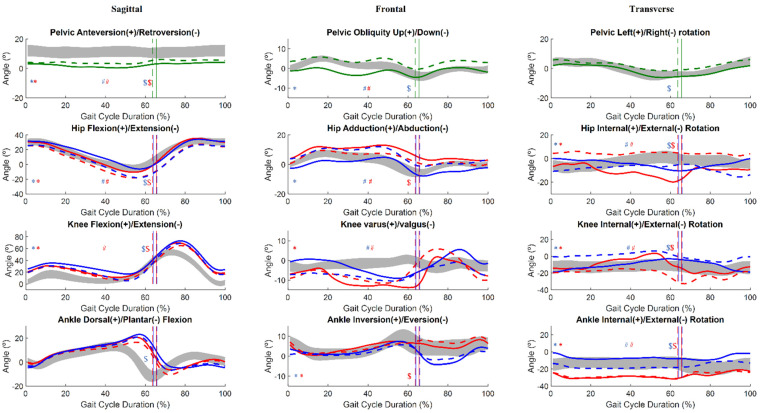
Average pelvis and lower limbs joint angles (in degrees) in the three anatomical planes during a gait cycle of the right (blue), left (red) lower limb and irrespective of the side (green). The joint angles at T0 are denoted by a solid line, while T1 is represented by a dashed line. The reference interval from the healthy gait dataset is shaded grey. A set of symbols are used to denote statistically significant differences, associated with large effect size, between T0 and T1 at the heel strike (*), midstance (#) and toe-off ($), with their color indicating the limb presenting such differences.

**Figure A5:**
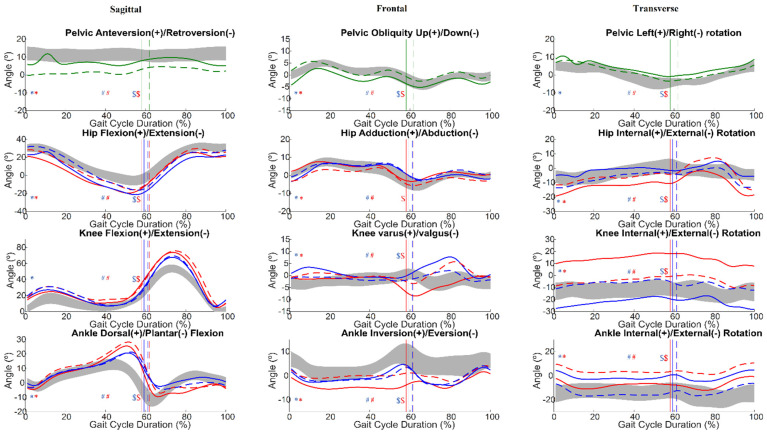
Average pelvis and lower limbs joint angles (in degrees) in the three anatomical planes during a gait cycle of the right (blue), left (red) lower limb and irrespective of the side (green). The joint angles at T0 are denoted by a solid line, while T1 is represented by a dashed line. The reference interval from the healthy gait dataset is shaded grey. A set of symbols are used to denote statistically significant differences, associated with large effect size, between T0 and T1 at the heel strike (*), midstance (#) and toe-off ($), with their color indicating the limb presenting such differences.

## Data Availability

Not applicable.

## Appendix A. Spatiotemporal Analysis

### Appendix A.1. Participant 1

**Table A1:** Spatiotemporal gait parameters results for T0 and T1, left (LLL) and right (RLL) lower limbs.

Parameter	T0		T1	
	LLL	RLL	LLL	RLL
**Speed (m/s)**	0.955		0.826	
**Stride width (m)**	0.156 ± 0.021		0.156 ± 0.025	
**Stride length (m)**	1.088 ± 0.034		0.995 ± 0.038	
**Step length (m)**	0.529 ± 0.023	0.559 ± 0.019	0.487 ± 0.029	0.503 ± 0.020
**Double Limb Support (s)**	0.158 ± 0.026	0.157 ± 0.013	0.160 ± 0.016	0.172 ± 0.027
**Cycle Time (s)**	1.137 ± 0.053	1.143 ± 0.084	1.183 ± 0.04	1.225 ± 0.071
**Stance Time (%)**	63.6	64.3	66.3	69.3
**Swing Time (%)**	36.3	36.2	37.7	38.4
**Cadence (steps/min)**	104.163 ± 5.926	106.346 ± 6.943	97.782 ± 7.512	100.591 ± 5.446

### Appendix A.2. Participant 2

**Table A2:** Spatiotemporal gait parameters results for T0 and T1, left (LLL) and right (RLL) lower limbs.

Parameter	T0		T1	
	LLL	RLL	LLL	RLL
**Speed (m/s)**	0.944		0.788	
**Stride width (m)**	0.093 ± 0.046		0.108 ± 0.045	
**Stride length (m)**	1.166 ± 0.047		1.01 ± 0.046	
**Step length (m)**	0.523 ± 0.022	0.634 ± 0.038	0.476 ± 0.033	0.524 ± 0.031
**Double Limb Support (s)**	0.129 ± 0.014	0.122 ± 0.016	0.166 ± 0.021	0.161 ± 0.025
**Cycle Time (s)**	1.239 ± 0.047	1.228 ± 0.047	1.28 ± 0.044	1.281 ± 0.057
**Stance Time (%)**	61.4	59.5	61.9	62.7
**Swing Time (%)**	38.6	40.6	38.1	37.4
**Cadence (steps/min)**	99.813 ± 4.258	93.951 ± 5.085	93.245 ± 5.459	93.089 ± 4.858

### Appendix A.3. Participant 3

**Table A3:** Spatiotemporal gait parameters results for T0 and T1, left (LLL) and right (RLL) lower limbs.

Parameter	T0		T1	
	LLL	RLL	LLL	RLL
**Speed (m/s)**	0.854		0.860	
**Stride width (m)**	0.104 ± 0.019		0.121 ± 0.022	
**Stride length (m)**	0.959 ± 0.038		0.926 ± 0.100	
**Step length (m)**	0.463 ± 0.024	0.493 ± 0.024	0.431 ± 0.108	0.482 ± 0.043
**Double Limb Support (s)**	0.151 ± 0.025	0.154 ± 0.030	0.169 ± 0.044	0.149 ± 0.025
**Cycle Time (s)**	1.128 ± 0.072	1.119 ± 0.053	1.079 ± 0.167	1.074 ± 0.050
**Stance Time (%)**	67.5	65.2	73.4	74.8
**Swing Time (%)**	42.4	44.6	45.2	44.6
**Cadence (steps/min)**	105.897 ± 6.684	107.852 ± 7.452	116.099 ± 28.739	107.825 ± 11.058

### Appendix A.4. Participant 4

**Table A4:** Spatiotemporal gait parameters results for T0 and T1, left (LLL) and right (RLL) lower limbs.

Parameter	T0		T1	
	LLL	RLL	LLL	RLL
**Speed (m/s)**	0.669		0.757	
**Stride width (m)**	0.177 ± 0.026		0.165 ± 0.030	
**Stride length (m)**	0.769 ± 0.046		0.845 ± 0.112	
**Step length (m)**	0.371 ± 0.033	0.396 ± 0.030	0.416 ± 0.036	0.434 ± 0.019
**Double Limb Support (s)**	0.158 ± 0.025	0.167 ± 0.041	0.161 ± 0.025	0.159 ± 0.036
**Cycle Time (s)**	1.148 ± 0.078	1.151 ± 0.070	1.134 ± 0.054	1.103 ± 0.197
**Stance Time (%)**	65.5	64.0	63.6	65.8
**Swing Time (%)**	36.9	36.0	36.4	37.0
**Cadence (steps/min)**	102.715 ± 8.615	105.822 ± 8.256	106.139 ± 8.520	105.713 ± 7.669

### Appendix A.5. Participant 5

**Table A5:** Spatiotemporal gait parameters results for T0 and T1, left (LLL) and right (RLL) lower limbs.

Parameter	T0		T1	
	LLL	RLL	LLL	RLL
**Speed (m/s)**	0.995		0.939	
**Stride width (m)**	0.093 ± 0.022		0.117 ± 0.026	
**Stride length (m)**	1.160 ± 0.032		1.167 ± 0.064	
**Step length (m)**	0.593 ± 0.016	0.561 ± 0.027	0.574 ± 0.059	0.585 ± 0.027
**Double Limb Support (s)**	0.089 ± 0.024	0.117 ± 0.013	0.123 ± 0.030	0.157 ± 0.026
**Cycle Time (s)**	1.157 ± 0.033	1.170 ± 0.025	1.222 ± 0.053	1.254 ± 0.061
**Stance Time (%)**	58.1	59.2	61.8	61.2
**Swing Time (%)**	41.9	40.8	38.2	38.8
**Cadence (steps/min)**	98.711 ± 3.223	106.280 ± 4.570	93.612 ± 7.640	98.656 ± 4.491

## Appendix B. Ground Reaction Forces

### Appendix B.1. Participant 1

**Table A6:** Mean and standard deviation of ground reaction forces in different gait cycle events at T0 and T1 for the left (LLL) and right (RLL) lower limbs, presented as % of body weight. * indicates a statistically significant difference (*p* < 0.05) between T0 and T1.

Parameter (% Body Weight)	T0		T1		Effect Size	
	LLL	RLL	LLL	RLL	LLL	RLL
FA1	−14.67 ± 2.26	−16.76 ± 1.96	−13.32 ± 1.35	−10.42 ± 1.73	0.27	0.83 *
FA3	14.96 ± 2.06	15.67 ± 1.82	9.58 ± 1.58	13.04 ± 1.66	0.83 *	0.69 *
FM1	−5.41 ± 1.77	−7.06 ± 1.84	−3.25 ± 1.27	−2.84 ± 1.06	0.50 *	0.76 *
FM2	6.38 ± 1.43	7.22 ± 0.66	6.49 ± 1.38	5.43 ± 1.46	0.08	0.54 *
FM3	9.13 ± 1.62	11.09 ± 0.87	9.66 ± 1.31	9.94 ± 3.22	0.01	0.20
FV1	115.49 ± 6.12	120.25 ± 4.67	113.45 ± 5.64	108.17 ± 5.54	0.08	0.72 *
FV2	74.15 ± 6.63	72.83 ± 4.98	78.86 ± 4.15	83.6 ± 2.77	0.24	0.77 *
FV3	102.34 ± 7.55	113.24 ± 3.69	105.43 ± 3.18	113.74 ± 3.92	0.60 *	0.00

### Appendix B.2. Participant 2

**Table A7:** Mean and standard deviation of ground reaction forces in different gait cycle events at T0 and T1 for the left (LLL) and right (RLL) lower limbs, presented as % of body weight. * indicates a statistically significant difference (*p* < 0.05) between T0 and T1.

Parameter (% Body Weight)	T0		T1		Effect Size	
	LLL	RLL	LLL	RLL	LLL	RLL
FA1	−25.64 ± 6.3	−27.48 ± 5.38	−25.01 ± 3.27	−21.52 ± 4.06	0.10	0.60 *
FA3	21.12 ± 1.64	20.75 ± 1.74	18.25 ± 1.15	19.57 ± 0.8	0.03 *	0.10
FM1	−5.29 ± 1.97	−6.4 ± 2.61	−3.94 ± 2.02	−3.33 ± 1.73	0.34	0.50 *
FM2	6.66 ± 3.53	5.78 ± 2.48	6.1 ± 3.22	5.88 ± 2.85	0.10	0.00
FM3	5.28 ± 1.56	5.99 ± 1.77	5.78 ± 2.58	4.98 ± 1.65	0.00	0.13
FV1	131.65 ± 4.32	127.51 ± 8.19	136.91 ± 4.76	138.01 ± 6.49	0.34	0.40 *
FV2	68.47 ± 5.82	68.56 ± 6.82	73.63 ± 5.93	71.6 ± 7.95	0.33	0.06
FV3	111.74 ± 7.44	107.22 ± 6.04	107.93 ± 2.59	112.05 ± 3.16	0.04	0.06

### Appendix B.3. Participant 3

**Table A8:** Mean and standard deviation of ground reaction forces in different gait cycle events at T0 and T1 for the left (LLL) and right (RLL) lower limbs, presented as % of body weight. * indicates a statistically significant difference (*p* < 0.05) between T0 and T1.

Parameter (% Body Weight)	T0		T1		Effect Size	
	LLL	RLL	LLL	RLL	LLL	RLL
FA1	−12.9 ± 1.9	−11.8 ± 1.6	−13.9 ± 2.3	−15.8 ± 2.9	0.1	0.1
FA3	12.1 ± 2.1	14.6 ± 2.6	15.5 ± 1.6	15.8 ± 1.2	0.0	0.0
FM1	−3.6 ± 1.3	−4.2 ± 1.7	−3.4 ± 1.5	−5.4 ± 1.1	0.3	0.2
FM2	6.2 ± 1.3	6.0 ± 1.1	7.2 ± 1.5	7.7 ± 1.7	0.1	0.1
FM3	6.8 ± 1.0	6.8 ± 0.9	7.4 ± 1.2	7.0 ± 1.4	0.3 *	0.1
FV1	111.6 ± 4.9	107.8 ± 4.5	117.4 ± 5.7	116.9 ± 5.1	0.3 *	0.1
FV2	76.5 ± 4.3	78.6 ± 2.7	75.0 ± 2.7	75.6 ± 3.7	0.5 *	0.3
FV3	108.2 ± 3.2	110.6 ± 4.1	113.7 ± 5.1	113.3 ± 5.5	0.8 *	0.3

### Appendix B.4. Participant 4

**Table A9:** Mean and standard deviation of ground reaction forces in different gait cycle events at T0 and T1 for the left (LLL) and right (RLL) lower limbs, presented as % of body weight. * indicates a statistically significant difference (*p* < 0.05) between T0 and T1.

Parameter (% body weight)	T0		T1		Effect Size	
	LLL	RLL	LLL	RLL	LLL	RLL
FA1	−17.5 ± 4.4	−17.2 ± 3.2	−14.5 ± 3.2	−17.1 ± 2.0	0.16	0.00
FA3	12.6 ± 2.4	14.6 ± 1.9	14.0 ± 1.3	11.9 ± 1.6	0.29	0.56 *
FM1	−2.3 ± 3.7	−3.5 ± 1.3	−1.4 ± 1.4	−3.33 ± 1.7	0.00	0.00
FM2	10.7 ± 1.4	11.4 ± 1.4	10.6 ± 1.3	10.7 ± 2.9	0.04	0.05
FM3	9.9 ± 1.6	9.7 ± 1.3	9.4 ± 1.5	9.9 ± 1.7	0.06	0.03
FV1	107.0 ± 10.1	116.0 ± 3.9	105.0 ± 5.0	109.7 ± 4.4	0.00	0.54 *
FV2	95.6 ± 6.2	92.7 ± 4.1	85.2 ± 2.6	85.3 ± 3.7	0.69 *	0.66 *
FV3	103.7 ± 5.9	103.3 ± 3.5	108.1 ± 4.6	98.8 ± 6.6	−0.40	−0.50 *

### Appendix B.5. Participant 5

**Table A10:** Mean and standard deviation of ground reaction forces in different gait cycle events at T0 and T1 for the left (LLL) and right (RLL) lower limbs, presented as % of body weight. * indicates a statistically significant difference (*p* < 0.05) between T0 and T1.

Parameter (% Body Weight)	T0		T1		Effect Size	
	LLL	RLL	LLL	RLL	LLL	RLL
FA1	−17.8 ± 4.8	−16.9 ± 3.3	−17.6 ± 2.0	−17.9 ± 3.6	0.02	0.03
FA3	17.3 ± 1.8	17.3 ± 2.7	15.0 ± 1.5	17.0 ± 0.9	0.61 *	0.00
FM1	−7.5 ± 2.3	−7.6 ± 2.8	−4.7 ± 1.2	−8.0 ± 2.2	−0.58 *	0.03
FM2	4.8 ± 1.4	5.4 ± 1.5	6.2 ± 1.0	5.0 ± 1.1	0.29 *	0.03
FM3	7.5 ± 1.9	7.6 ± 1.9	6.5 ± 0.9	8.8 ± 1.1	0.21	0.25
FV1	113.9 ± 4.2	114.2 ± 4.8	114.0 ± 4.1	116.2 ± 3.7	0.00	0.17
FV2	79.6 ± 4.1	78.2 ± 4.3	80.1 ± 4.1	79.1 ± 2.9	0.03	0.03
FV3	112.4 ± 8.2	113.6 ± 6.3	110.2 ± 5.1	104.5 ± 35.9	−0.24	−0.16

## Appendix C. Angular Kinematics

### Appendix C.1. Participant 1

**Table A11:** Sagittal plane mean and standard deviation angles of different joints (hip, pelvis, knee and ankle), at T0 and T1, at the heel strike (HS), mid stance (MD), and toe off (TO), for both lower limbs, along with the *p*-value between both assessment periods. The effect size is presented as large (^a^), moderate (^b^) or small (^c^).

Joint Angle (Degrees)	Time of Assessment	Left Lower Limb			Right Lower Limb		
		HS	MD	TO	HS	MD	TO
Pelvis	T0	4.07 ± 0.86	−0.55 ± 0.83	6.57 ± 0.64	−2.08 ± 1.02	1.31 ± 1.06	−4.85 ± 0.76
	T1	3.62 ± 1.15	0.57 ± 0.49	6.83 ± 0.78	0.26 ± 1.55	1.16 ± 0.84	−3.78 ± 0.80
	*p*-value	0.016 ^a^	0.011 ^a^	0.213 ^b^	0.000 ^a^	0.305 ^c^	0.003 ^a^
Left Hip	T0	20.31 ± 1.98	1.26 ± 1.87	−5.11 ± 2.44	−11.56 ± 1.21	21.09 ± 7.36	19.98 ± 2.08
	T1	21.36 ± 2.10	0.23 ± 1.58	−2.98 ± 2.95	−10.38 ± 2.20	24.98 ± 1.65	17.91 ± 3.05
	*p*-value	0.083 ^a^	0.083 ^a^	0.022 ^a^	0.134 ^b^	0.000 ^a^	0.011 ^a^
Right Hip	T0	−9.83 ± 1.36	21.55 ± 1.97	21.47 ± 2.33	25.93 ± 1.28	3.98 ± 5.54	−4.28 ± 1.9
	T1	−11.09 ± 1.45	20.76 ± 2.90	17.81 ± 3.39	24.43 ± 3.20	−0.46 ± 1.76	−5.17 ± 2.23
	*p*-value	0.025 ^b^	0.334 ^c^	0.001 ^a^	0.016 ^b^	0.000 ^a^	0.691 ^c^
Left Knee	T0	7.31 ± 1.47	12.4 ± 1.9	43.56 ± 3.83	10.15 ± 0.48	59.47 ± 11.38	22.08 ± 3.77
	T1	7.60 ± 1.50	8.05 ± 2.32	40.56 ± 4.44	7.11 ± 1.33	60.85 ± 2.03	17.84 ± 3.31
	*p*-value	0.697 ^c^	0.000 ^a^	0.008 ^b^	0.000 ^a^	0.741 ^c^	0.001 ^a^
Right Knee	T0	15.09 ± 1.58	53.61 ± 1.42	25.47 ± 2.99	12.52 ± 1.38	16.85 ± 3.05	40.46 ± 5.38
	T1	9.01 ± 0.84	51.67 ± 4.96	18.4 ± 4.26	10.58 ± 3.32	8.81 ± 1.93	35.48 ± 5.14
	*p*-value	0.000 ^a^	0.460 ^c^	0.000 ^a^	0.030 ^b^	0.000 ^a^	0.639 ^c^
Left Ankle	T0	−3.86 ± 0.97	8.56 ± 0.91	7.08 ± 3.31	20.2 ± 0.74	−2.45 ± 5.37	1.69 ± 1.76
	T1	−3.31 ± 1.53	9.57 ± 1.10	8.74 ± 2.53	21.58 ± 1.05	−1.89 ± 1.50	2.42 ± 1.70
	*p*-value	0.247 ^c^	0.000 ^a^	0.068 ^b^	0.017 ^b^	0.073 ^b^	0.017 ^b^
Right Ankle	T0	22.7 ± 1.87	7.19 ± 0.59	3.34 ± 1.55	3.37 ± 0.79	10.21 ± 2.05	12.01 ± 2.84
	T1	20.45 ± 1.68	6.54 ± 1.30	2.78 ± 2.29	0.26 ± 1.68	8.5 ± 1.72	12.63 ± 2.10
	*p*-value	0.000 ^a^	0.053 ^a^	0.768 ^c^	0.000 ^a^	0.006 ^b^	0.427 ^c^

**Table A12:** Frontal plane mean and standard deviation angles of different joints (hip, pelvis, knee and ankle), at T0 and T1, at the heel strike (HS), mid stance (MD), and toe off (TO), for both lower limbs, along with the *p*-value between both assessment periods. The effect size is presented as large (^a^), moderate (^b^) or small (^c^).

Joint Angle (Degrees)	Time of Assessment	Left Lower Limb			Right Lower Limb		
		HS	MD	TO	HS	MD	TO
Pelvis	T0	−2.07 ± 1.17	−2.48 ± 1.73	−2.96 ± 1.34	0.23 ± 1.02	−4.57 ± 1.98	−1.62 ± 1.51
	T1	−0.01 ± 1.31	−0.48 ± 1.21	0.19 ± 2.78	2.59 ± 1.75	−2.24 ± 1.87	0.24 ± 1.82
	*p*-value	0.000 ^a^	0.001 ^a^	0.000 ^a^	0.000 ^a^	0.002 ^b^	0.047 ^b^
Left Hip	T0	−8.80 ± 1.30	1.37 ± 0.98	−11.91 ± 1.02	−0.28 ± 1	−7.28 ± 2.07	1.71 ± 1.62
	T1	−7.61 ± 1.49	0.73 ± 0.98	−12.27 ± 1.49	−1.34 ± 1.59	−7.41 ± 1.13	2.20 ± 1.36
	*p*-value	0.000 ^a^	0.234 ^a^	0.147 ^a^	0.009 ^b^	0.244 ^a^	0.338 ^b^
Right Hip	T0	6.57 ± 1.05	−0.70 ± 1.04	7.99 ± 1.54	−4.97 ± 1.90	6.06 ± 3.15	−2.99 ± 1.06
	T1	5.51 ± 1.53	−2.25 ± 1.16	6.96 ± 1.35	−4.66 ± 2.01	4.36 ± 1.62	−4.39 ± 1.32
	*p*-value	0.007 ^a^	0.003 ^c^	0.042 ^b^	0.715 ^c^	0.006 ^c^	0.046 ^a^
Left Knee	T0	0.60 ± 0.47	−0.27 ± 0.48	−8.66 ± 1.03	−1.01 ± 0.69	−5.76 ± 1.76	1.95 ± 2.43
	T1	1.13 ± 0.60	0.77 ± 0.55	−3.08 ± 1.18	0.46 ± 0.40	2.13 ± 1.74	1.50 ± 1.10
	*p*-value	0.001 ^a^	0.000 ^a^	0.254 ^a^	0.000 ^a^	0.254 ^a^	0.733 ^c^
Right Knee	T0	2.98 ± 1.41	4.07 ± 2.26	2.36 ± 2.05	−1.67 ± 0.89	−0.56 ± 1.06	−2.40 ± 2.18
	T1	−1.22 ± 0.67	0.38 ± 1.54	−0.89 ± 1.06	−0.79 ± 1.82	−0.71 ± 0.63	−3.48 ± 1.03
	*p*-value	0.254 ^b^	0.001 ^a^	0.000 ^a^	0.217 ^c^	0.099 ^c^	0.069 ^b^
Left Ankle	T0	7.71 ± 1.04	1.62 ± 1.04	9.07 ± 1.21	7.85 ± 0.95	7.40 ± 0.72	−1.74 ± 0.51
	T1	6.65 ± 1.05	−0.54 ± 1.67	5.42 ± 1.93	3.41 ± 1.22	6.74 ± 1.04	−3.29 ± 1.31
	*p*-value	0.002 ^a^	0.001 ^a^	0.000 ^a^	0.000 ^a^	0.027 ^b^	0.000 ^a^
Right Ankle	T0	3.01 ± 1.23	7.19 ± 0.76	−1.77 ± 0.99	6.36 ± 1.05	−0.26 ± 2.13	8.27 ± 1.70
	T1	1.07 ± 1.31	1.43 ± 0.89	−2.66 ± 1.37	5.18 ± 1.37	−1.17 ± 1.32	3.00 ± 1.18
	*p*-value	0.002 ^a^	0.000 ^a^	0.130 ^b^	0.004 ^a^	0.455 ^c^	0.000 ^a^

**Table A13:** Transverse plane mean and standard deviation angles of different joints (hip, pelvis, knee and ankle), at T0 and T1, at the heel strike (HS), mid stance (MD), and toe off (TO), for both lower limbs, along with the *p*-value between both assessment periods. The effect size is presented as large (^a^), moderate (^b^) or small (^c^).

Joint Angle (Degrees)	Time of Assessment	Left Lower Limb			Right Lower Limb		
		HS	MD	TO	HS	MD	TO
Pelvis	T0	80.93 ± 2.86	89.49 ± 1.12	98.11 ± 2.35	100.6 ± 2.45	90.25 ± 3.55	82.35 ± 2.64
	T1	83.66 ± 2.87	89.69 ± 2.64	96.84 ± 3.09	98.55 ± 3.08	91.15 ± 2.88	83.64 ± 2.83
	*p*-value	0.000 ^a^	0.691 ^c^	0.050 ^b^	0.019 ^b^	0.322 ^c^	0.360 ^b^
Left Hip	T0	−23.28 ± 2.89	−19.29 ± 1.27	−16.00 ± 2.73	−19.69 ± 1.42	−15.07 ± 1.91	−15.77 ± 5.17
	T1	−19.3 ± 2.52	−14.91 ± 2.65	−11.03 ± 2.96	−15.01 ± 2.52	−6.70 ± 2.25	−14.15 ± 2.61
	*p*-value	0.001 ^a^	0.000 ^a^	0.000 ^a^	0.000 ^a^	0.000 ^a^	0.434 ^b^
Right Hip	T0	−1.85 ± 3.12	−1.30 ± 3.03	−1.29 ± 3.38	−10.04 ± 2.16	−0.45 ± 4.58	−0.58 ± 4.13
	T1	−11.79 ± 2.46	−10.74 ± 3.39	−14.44 ± 2.84	−22.56 ± 3.21	−13.85 ± 2.76	−8.82 ± 2.55
	*p*-value	0.001 ^b^	0.001 ^a^	0.000 ^a^	0.000 ^a^	0.000 ^a^	0.003 ^a^
Left Knee	T0	−7.00 ± 3.02	2.24 ± 1.69	−4.02 ± 4.30	10.41 ± 3.19	−6.85 ± 4.43	−7.45 ± 3.09
	T1	−3.00 ± 2.52	4.34 ± 3.04	5.29 ± 2.93	9.51 ± 2.32	2.54 ± 1.30	−0.74 ± 2.70
	*p*-value	0.000 ^a^	0.009 ^b^	0.000 ^a^	0.114 ^c^	0.000 ^b^	0.001 ^a^
Right Knee	T0	15.09 ± 1.58	−25.87 ± 2.23	−22.64 ± 4.00	−16.74 ± 1.99	−12.43 ± 2.75	−18.55 ± 7.88
	T1	7.42 ± 2.14	−0.95 ± 3.71	0.48 ± 3.87	0.21 ± 3.52	4.18 ± 2.18	1.46 ± 2.24
	*p*-value	0.000 ^a^	0.052 ^a^	0.000 ^a^	0.000 ^a^	0.001 ^a^	0.001 ^b^
Left Ankle	T0	10.81 ± 2.34	7.44 ± 1.98	15.45 ± 2.39	9.9 ± 2.02	16.11 ± 2.14	5.16 ± 2.27
	T1	5.66 ± 1.17	2.59 ± 2.60	4.75 ± 2.04	6.55 ± 1.84	4.91 ± 1.06	−1.14 ± 2.31
	*p*-value	0.000 ^a^	0.001 ^a^	0.000 ^a^	0.000 ^a^	0.000 ^a^	0.000 ^a^
Right Ankle	T0	−0.85 ± 1.37	10.52 ± 1.85	−1.91 ± 1.46	−0.88 ± 1.18	−3.22 ± 1.56	6.37 ± 5.38
	T1	−12.64 ± 2.96	−13.59 ± 1.73	−18.66 ± 3.13	−9.70 ± 1.89	−15.06 ± 2.78	−13.00 ± 2.27
	*p*-value	0.000 ^a^	0.001 ^a^	0.000 ^a^	0.000 ^a^	0.000 ^a^	0.000 ^a^

P1 shows a more retroverted pelvis than the reference population, although with a normal angle variation during the whole gait cycle. At T1 this angle was lower in general, being even lower than the reference population. In the frontal plane, the pelvic obliquity during the RLL cycle is higher than the reference data. In the transverse plane left and right rotations are more prominent at T1 than T0 and the variation is around −10 to 10 degrees. P1 also presents a delayed toe-off at T1, more prominent for the right lower limb cycles, as can be seen in the charts showing the angular behavior of the other limbs. The hip angle shows higher extension than the reference population, more abduction (frontal plane) and external rotation (transverse plane) at T1 than T0. It also presents an abduction lower than the reference data for left lower limb cycles, in both assessments, at T1 with a frontal range of motion of 25 degrees. Looking at the transverse plane, all except right lower limb cycles during T0 show more external rotation (up to 15° lower) during the whole gait cycle, than the reference data. The stance phase knee flexion is higher at T1 than T0 and also than the reference. Swing phase knee flexion is higher than the reference gait data and higher at T1 than T0. The knee and ankle rotation are more internal at T1 and the transition from dorsi- to plantar-flexion occurs later than in the reference population. In the transverse plane, knee rotation is in general more internal than the reference data, except for the right lower limb cycles at T0 which is mainly within a normal range. The ankle angle shows a higher dorsiflexion than the reference data, before the toe-off. In the frontal plane, the first eversion is also more prominent than the correspondent for the reference. In the transverse plane it is possible to see that with the exception of right lower limb cycles at T1, all assessments show higher internal rotation of the ankle during the whole gait cycle.

### Appendix C.2. Participant 2

**Table A14:** Sagittal plane mean and standard deviation angles of different joints (hip, pelvis, knee and ankle), at T0 and T1, at the heel strike (HS), mid stance (MD), and toe off (TO), for both lower limbs, along with the *p*-value between both assessment periods. The effect size is presented as large (^a^), moderate (^b^) or small (^c^).

Joint Angle (Degrees)	Time of Assessment	Left Lower Limb			Right Lower Limb		
		HS	MD	TO	HS	MD	TO
Pelvis	T0	6.53 ± 0.98	5.67 ± 0.95	2.80 ± 0.86	−2.10 ± 0.89	−1.43 ± 1.01	0.84 ± 1.35
	T1	5.94 ± 1.01	3.74 ± 0.89	1.74 ± 1.00	−3.77 ± 1.35	−1.55 ± 1.04	−0.07 ± 1.37
	*p*-value	0.005 ^a^	0.000 ^a^	0.004 ^b^	0.000 ^a^	0.943 ^c^	0.003 ^b^
Left Hip	T0	27.92 ± 1.70	12.99 ± 1.67	−8.20 ± 1.78	−10.18 ± 2.69	40.50 ± 1.85	30.04 ± 2.02
	T1	24.27 ± 2.15	8.75 ± 1.83	−3.89 ± 2.97	−14.68 ± 2.83	35.52 ± 1.92	20.76 ± 5.80
	*p*-value	0.000 ^a^	0.000 ^a^	0.000 ^a^	0.000 ^a^	0.000 ^a^	0.000 ^a^
Right Hip	T0	−11.71 ± 2.13	37.42 ± 2.64	30.31 ± 2.08	27.06 ± 2.51	9.15 ± 1.82	−4.83 ± 2.17
	T1	−16.24 ± 2.07	33.62 ± 2.43	20.01 ± 2.25	19.29 ± 1.85	4.55 ± 1.58	−3.69 ± 4.28
	*p*-value	0.000 ^a^	0.000 ^a^	0.000 ^a^	0.000 ^a^	0.000 ^a^	0.601 ^c^
Left Knee	T0	15.81 ± 2.45	23.95 ± 3.40	43.61 ± 4.68	23.63 ± 4.41	77.63 ± 2.23	35.01 ± 2.68
	T1	15.36 ± 3.84	17.80 ± 2.02	47.76 ± 4.34	9.2 ± 3.05	73.84 ± 2.39	31.19 ± 5.12
	*p*-value	0.493 ^c^	0.000 ^a^	0.006 ^a^	0.000 ^a^	0.000 ^a^	0.000 ^a^
Right Knee	T0	−1.78 ± 0.84	75.87 ± 1.92	34.47 ± 3.29	15.91 ± 2.85	17.54 ± 2.24	37.75 ± 3.02
	T1	1.24 ± 1.95	70.13 ± 3.03	27.99 ± 3.14	10.23 ± 3.00	11.75 ± 2.83	42.93 ± 7.45
	*p*-value	0.000 ^a^	0.000 ^a^	0.000 ^a^	0.000 ^a^	0.000 ^a^	0.000 ^a^
Left Ankle	T0	1.76 ± 1.75	28.12 ± 2.66	32.94 ± 2.77	47.60 ± 3.26	8.42 ± 2.11	21.92 ± 1.96
	T1	−4.47 ± 2.00	9.01 ± 1.90	6.68 ± 4.32	19.18 ± 2.96	−12.22 ± 3.30	8.97 ± 2.62
	*p*-value	0.000 ^a^	0.000 ^a^	0.000 ^a^	0.000 ^a^	0.000 ^a^	0.000 ^a^
Right Ankle	T0	20.36 ± 2.28	−13.29 ± 2.48	5.94 ± 1.58	−3.13 ± 1.24	10.61 ± 1.26	16.05 ± 1.83
	T1	16.87 ± 2.72	−11.05 ± 2.40	8.66 ± 2.39	−3.93 ± 1.65	−1.55 ± 1.04	8.36 ± 3.59
	*p*-value	0.000 ^a^	0.001 ^a^	0.000 ^a^	0.587 ^c^	0.000 ^a^	0.000 ^a^

**Table A15:** Frontal plane mean and standard deviation angles of different joints (hip, pelvis, knee and ankle), at T0 and T1, at the heel strike (HS), mid stance (MD), and toe off (TO), for both lower limbs, along with the *p*-value between both assessment periods. The effect size is presented as large (^a^), moderate (^b^) or small (^c^).

Joint Angle (Degrees)	Time of Assessment	Left Lower Limb			Right Lower Limb		
		HS	MD	TO	HS	MD	TO
Pelvis	T0	1.12 ± 1.20	0.22 ± 1.61	−1.63 ± 1.11	−1.10 ± 1.07	0.29 ± 1.72	0.28 ± 1.57
	T1	−0.55 ± 0.82	−0.67 ± 1.23	−2.56 ± 1.17	−2.10 ± 0.99	−1.63 ± 1.06	−2.68 ± 0.90
	*p*-value	0.000 ^a^	0.062 ^c^	0.088 ^c^	0.017 ^a^	0.000 ^a^	0.000 ^a^
Left Hip	T0	−8.06 ± 1.63	−1.96 ± 1.68	−2.70 ± 2.23	0.68 ± 2.11	2.87 ± 1.34	0.75 ± 1.98
	T1	−6.92 ± 2.30	−1.90 ± 2.09	−3.31 ± 2.21	1.67 ± 2.71	0.81 ± 1.95	1.42 ± 2.08
	*p*-value	0.011 ^a^	0.833 ^c^	0.902 ^c^	0.073 ^b^	0.000 ^a^	0.115 ^b^
Right Hip	T0	6.37 ± 2.04	−0.81 ± 1.02	0.63 ± 1.79	−7.76 ± 1.83	0.94 ± 2.26	1.34 ± 1.48
	T1	6.60 ± 1.89	−1.83 ± 1.53	−0.54 ± 2.48	−8.90 ± 2.25	−0.41 ± 2.49	−0.55 ± 2.56
	*p*-value	0.853 ^a^	0.075 ^c^	0.285 ^c^	0.163 ^b^	0.019 ^b^	0.001 ^b^
Left Knee	T0	−2.95 ± 1.42	−3.45 ± 0.96	−6.57 ± 2.42	−3.62 ± 1.73	−6.39 ± 2.34	−3.03 ± 1.89
	T1	1.34 ± 1.11	1.34 ± 1.00	−1.58 ± 2.51	1.87 ± 0.94	9.49 ± 3.96	2.80 ± 2.01
	*p*-value	0.001 ^a^	0.000 ^a^	0.003 ^a^	0.000 ^a^	0.003 ^a^	0.000 ^a^
Right Knee	T0	−2.53 ± 1.50	12.12 ± 2.20	7.41 ± 2.26	2.34 ± 0.96	3.69 ± 0.83	5.99 ± 1.47
	T1	4.88 ± 1.40	12.05 ± 2.66	5.15 ± 1.96	2.38 ± 1.09	4.48 ± 1.11	6.51 ± 4.41
	*p*-value	0.003 ^b^	0.361 ^c^	0.000 ^a^	0.361 ^c^	0.093 ^c^	0.575 ^c^
Left Ankle	T0	1.72 ± 1.81	−10.04 ± 1.08	−3.13 ± 1.30	−7.72 ± 1.63	−5.15 ± 4.05	−10.37 ± 1.30
	T1	7.36 ± 1.77	4.00 ± 1.01	8.75 ± 1.22	11.47 ± 1.52	5.20 ± 2.69	3.50 ± 1.88
	*p*-value	0.000 ^a^	0.000 ^a^	0.000 ^a^	0.000 ^a^	0.000 ^a^	0.000 ^a^
Right Ankle	T0	3.83 ± 1.98	3.12 ± 2.78	−3.51 ± 1.19	5.03 ± 1.82	−1.98 ± 1.35	5.62 ± 0.57
	T1	5.48 ± 1.88	7.48 ± 2.76	−3.05 ± 1.93	5.95 ± 2.54	−1.63 ± 1.06	3.39 ± 1.92
	*p*-value	0.003 ^b^	0.000 ^a^	0.463 ^c^	0.009 ^a^	0.143 ^c^	0.000 ^a^

**Table A16:** Transverse plane mean and standard deviation angles of different joints (hip, pelvis, knee and ankle), at T0 and T1, at the heel strike (HS), mid stance (MD), and toe off (TO), for both lower limbs, along with the *p*-value between both assessment periods. The effect size is presented as large (^a^), moderate (^b^) or small (^c^).

Joint Angle (Degrees)	Time of Assessment	Left Lower Limb			Right Lower Limb		
		HS	MD	TO	HS	MD	TO
Pelvis	T0	80.91 ± 2.70	92.93 ± 2.71	100.20 ± 2.53	106.72 ± 3.43	94.48 ± 2.71	85.78 ± 2.60
	T1	82.6 ± 2.62	90.43 ± 2.79	94.28 ± 2.91	98.19 ± 3.48	92.22 ± 3.37	87.3 ± 2.86
	*p*-value	0.001 ^b^	0.009 ^c^	0.000 ^a^	0.000 ^a^	0.004 ^b^	0.029 ^a^
Left Hip	T0	−16.47 ± 4.22	−9.95 ± 2.08	−5.65 ± 3.27	−5.51 ± 4.01	−2.83 ± 2.74	−9.63 ± 2.45
	T1	−5.29 ± 3.63	−2.9 ± 2.52	0.54 ± 2.89	2.86 ± 3.82	10.58 ± 4.37	−2.21 ± 2.62
	*p*-value	0.000 ^a^	0.000 ^a^	0.000 ^a^	0.000 ^a^	0.000 ^a^	0.000 ^a^
Right Hip	T0	1.92 ± 4.55	3.28 ± 2.70	−1.59 ± 3.21	−9.75 ± 3.28	−0.48 ± 3.89	1.98 ± 3.18
	T1	−1.64 ± 3.13	2.62 ± 2.74	−10.83 ± 3.35	−22.34 ± 3.15	−7.56 ± 3.42	−1.79 ± 7.59
	*p*-value	0.001 ^b^	0.072 ^c^	0.000 ^a^	0.000 ^a^	0.000 ^a^	0.000 ^b^
Left Knee	T0	−1.49 ± 3.35	2.65 ± 2.32	−4.43 ± 4.59	9.53 ± 4.26	−10.71 ± 5.35	−4.58 ± 3.63
	T1	−34.33 ± 3.53	−22.25 ± 2.00	−26.19 ± 2.37	−21.55 ± 2.69	−29.86 ± 3.74	−25.85 ± 2.97
	*p*-value	0.000 ^a^	0.000 ^a^	0.000 ^a^	0.000 ^a^	0.000 ^b^	0.000 ^a^
Right Knee	T0	8.29 ± 1.67	−18.79 ± 2.99	−29.62 ± 3.14	−28.71 ± 3.42	−23.09 ± 1.9	−30.18 ± 3.10
	T1	−25.11 ± 2.41	−25.39 ± 4.18	−24.07 ± 2.52	−25.85 ± 3.25	−25.74 ± 1.61	−32.97 ± 7.68
	*p*-value	0.000 ^a^	0.000 ^a^	0.000 ^a^	0.004 ^b^	0.000 ^b^	0.067 ^c^
Left Ankle	T0	1.48 ± 1.51	6.18 ± 1.36	10.73 ± 1.84	6.07 ± 1.55	11.9 ± 4.68	5.21 ± 2.39
	T1	19.63 ± 2.81	8.91 ± 1.35	15.81 ± 3.15	11.73 ± 1.31	21.33 ± 4.01	8.16 ± 1.46
	*p*-value	0.000 ^a^	0.000 ^a^	0.000 ^a^	0.000 ^a^	0.000 ^a^	0.000 ^a^
Right Ankle	T0	0.14 ± 2.08	15.31 ± 3.33	0.95 ± 1.45	4.76 ± 1.25	−2.09 ± 1.32	5.89 ± 2.80
	T1	0.94 ± 1.46	12.09 ± 3.81	−3.02 ± 1.55	10.3 ± 2.42	92.22 ± 3.37	3.43 ± 2.86
	*p*-value	0.057 ^c^	0.002 ^c^	0.000 ^a^	0.000 ^a^	0.000 ^a^	0.001 ^a^

P2 also shows a slightly delayed toe-off on the right lower limb cycles, at T1, and a statistically significant higher retroversion of the pelvis at HS at T1. In the frontal plane, the last elevations are higher than the reference data and at T1 they exceed the normal 5 degrees of variation, reaching near 10°. In the transverse plane left and right rotations are more prominent at T1 than T0 and the variation is around −10 to 20 degrees at T0 and lowers to −10 to 10 at T1. The hip is more extended and also more flexed (right after extension) than in the reference population and in the frontal plane shows a range of motion of 20 degrees. In the transverse plane the rotators pattern is closer to the reference values than P1 but with excessive rotation (more than 10 degrees total displacement). The stance phase knee flexion is higher at T1 than T0 and also than the reference population. Swing phase knee flexion is higher than the reference data and higher at T1 than T0 (steppage). In the transverse plane, knee rotation is more external than normal, reaching −40° at some point, except for the left lower limb cycles at T0 in which the knee is more internally rotated. The ankle angle shows a marked asymmetry with the left lower limb cycles, at T0, being more dorsiflexed in general (up to 45°), with a timid plantar-flexion right after toe-off. In the frontal plane it is possible to see that the eversion angles vary from the right limb cycles, and left limb cycles at T0 and T1, but the pattern is similar and the eversion is not marked. The swing phase was different for all the assessments. The transverse plane, as for P1, shows a more internally rotated ankle than the reference population, along the entire gait cycle.

### Appendix C.3. Participant 3

**Table A17:** Sagittal plane mean and standard deviation angles of different joints (hip, pelvis, knee and ankle), at T0 and T1, at the heel strike (HS), mid stance (MD), and toe off (TO), for both lower limbs, along with the *p*-value between both assessment periods. The effect size is presented as large (^a^), moderate (^b^) or small (^c^).

Joint Angle (Degrees)	Time of Assessment	Left Lower Limb			Right Lower Limb		
		HS	MD	TO	HS	HS	MD
Pelvis	T0	0.68 ± 0.84	0.28 ± 0.59	5.71 ± 0.69	2.12 ± 0.67	1.82 ± 0.69	−4.31 ± 0.71
	T1	0.60 ± 1.72	−0.79 ± 0.87	6.26 ± 1.13	0.89 ± 1.17	1.64 ± 0.87	−5.30 ± 0.77
	*p*-value	0.812 ^c^	0.000 ^a^	0.013 ^b^	0.000 ^a^	0.480 ^c^	0.000 ^a^
Left Hip	T0	24.31 ± 1.57	2.80 ± 0.89	−5.20 ± 2.26	−16.05 ± 1.51	23.84 ± 1.49	19.7 ± 3.26
	T1	23.45 ± 4.22	2.43 ± 2.22	−5.02 ± 5.11	−19.19 ± 2.50	22.80 ± 3.48	19.00 ± 3.20
	*p*-value	0.057 ^b^	0.057 ^b^	0.657 ^c^	0.000 ^a^	0.008 ^b^	0.302 ^c^
Right Hip	T0	−11.48 ± 4.40	25.41 ± 2.31	23.54 ± 2.98	27.85 ± 2.51	6.91 ± 3.45	0.47 ± 3.29
	T1	−18.07 ± 5.30	23.39 ± 2.27	20.85 ± 7.73	25.87 ± 3.12	2.20 ± 2.58	−5.90 ± 2.86
	*p*-value	0.000 ^a^	0.000 ^a^	0.004 ^b^	0.001 ^b^	0.000 ^a^	0.000 ^a^
Left Knee	T0	9.81 ± 2.38	4.35 ± 0.94	32.07 ± 4.94	−4.89 ± 2.2	46.2 ± 2.64	17.6 ± 3.92
	T1	12.89 ± 4.21	8.16 ± 1.55	34.92 ± 6.12	−8.55 ± 2.52	49.19 ± 5.58	22.59 ± 2.51
	*p*-value	0.002 ^b^	0.000 ^a^	0.035 ^c^	0.000 ^a^	0.001 ^b^	0.000 ^a^
Right Knee	T0	3.77 ± 1.03	49.62 ± 3.39	23.12 ± 3.92	15.3 ± 3.63	12.58 ± 5.45	40.2 ± 5.71
	T1	−8.02 ± 5.92	51.14 ± 3.63	21.04 ± 5.88	12.4 ± 4.49	6.88 ± 3.42	35.76 ± 5.27
	*p*-value	0.000 ^a^	0.087 ^c^	0.008 ^b^	0.001 ^b^	0.000 ^a^	0.001 ^b^
Left Ankle	T0	1.21 ± 1.30	6.61 ± 1.19	9.94 ± 3.13	14.11 ± 2.37	5.65 ± 0.77	2.58 ± 1.51
	T1	−0.90 ± 2.35	9.03 ± 1.14	12.8 ± 3.39	13.17 ± 2.38	6.97 ± 1.13	6.08 ± 1.79
	*p*-value	0.029 ^b^	0.000 ^a^	0.001 ^b^	0.000 ^a^	0.000 ^b^	0.000 ^a^
Right Ankle	T0	15.55 ± 5.38	7.45 ± 2.15	2.77 ± 1.34	1.81 ± 1.56	7.54 ± 1.86	11.97 ± 4.39
	T1	11.18 ± 3.08	8.58 ± 1.25	3.29 ± 2.38	−2.13 ± 2.41	6.83 ± 1.54	13.70 ± 2.41
	*p*-value	0.000 ^a^	0.012 ^b^	0.307 ^c^	0.000 ^a^	0.002 ^b^	0.155 ^c^

**Table A18:** Frontal plane mean and standard deviation angles of different joints (hip, pelvis, knee and ankle), at T0 and T1, at the heel strike (HS), mid stance (MD), and toe off (TO), for both lower limbs, along with the *p*-value between both assessment periods. The effect size is presented as large (^a^), moderate (^b^) or small (^c^).

Joint Angle (Degrees)	Time of Assessment	Left Lower Limb			Right Lower Limb		
		HS	MD	TO	HS	MD	TO
Pelvis	T0	2.55 ± 0.67	3.38 ± 0.81	2.61 ± 0.88	2.46 ± 1.01	3.10 ± 0.76	2.81 ± 0.76
	T1	0.37 ± 1.61	1.49 ± 2.17	1.80 ± 1.82	1.47 ± 1.47	0.98 ± 1.81	0.20 ± 2.75
	*p*-value	0.000 ^a^	0.000 ^b^	0.001 ^a^	0.000 ^b^	0.000 ^a^	0.000 ^b^
Left Hip	T0	1.36 ± 1.32	6.24 ± 0.70	−2.41 ± 0.85	2.36 ± 0.94	1.50 ± 0.74	9.19 ± 1.08
	T1	0.38 ± 2.23	6.29 ± 1.08	−4.07 ± 1.36	2.62 ± 1.40	0.36 ± 1.05	9.28 ± 1.13
	*p*-value	0.088 ^a^	0.044 ^b^	0.000 ^a^	0.334 ^a^	0.000 ^a^	0.558 ^b^
Right Hip	T0	5.79 ± 1.84	1.97 ± 0.79	10.02 ± 0.91	4.18 ± 0.92	8.31 ± 0.66	−1.12 ± 1.51
	T1	4.14 ± 1.80	1.40 ± 1.01	9.75 ± 1.39	2.30 ± 1.60	7.46 ± 1.14	−3.07 ± 1.32
	*p*-value	0.000 ^b^	0.018 ^c^	0.088 ^b^	0.000 ^a^	0.001 ^b^	0.000 ^a^
Left Knee	T0	−4.33 ± 0.52	−4.43 ± 0.39	−8.73 ± 0.99	−1.95 ± 1.02	−9.91 ± 1.07	−3.38 ± 1.61
	T1	−4.15 ± 0.86	−3.75 ± 0.69	−5.08 ± 0.65	−1.40 ± 0.92	−2.10 ± 1.05	−2.64 ± 1.01
	*p*-value	0.596 ^c^	0.000 ^a^	0.000 ^a^	0.011 ^c^	0.000 ^a^	0.046 ^c^
Right Knee	T0	−14.23 ± 3.12	5.47 ± 3.00	3.11 ± 2.07	−0.87 ± 1.48	−1.75 ± 1.16	5.82 ± 3.69
	T1	−1.54 ± 1.39	−3.11 ± 2.01	−1.66 ± 1.52	−3.49 ± 1.64	−3.07 ± 1.02	−2.85 ± 1.82
	*p*-value	0.000 ^a^	0.463 ^c^	0.000 ^a^	0.000 ^a^	0.075 ^b^	0.899 ^c^
Left Ankle	T0	1.12 ± 1.11	−0.95 ± 1.89	5.51 ± 1.28	−0.55 ± 1.37	3.18 ± 1.09	−1.68 ± 1.73
	T1	2.65 ± 1.69	−1.26 ± 0.82	5.78 ± 1.69	0.06 ± 0.85	3.71 ± 1.13	−2.12 ± 1.22
	*p*-value	0.973 ^c^	0.392 ^c^	0.436 ^c^	0.044 ^b^	0.026 ^c^	0.336 ^c^
Right Ankle	T0	2.41 ± 6.95	4.00 ± 1.93	−0.29 ± 2.31	3.58 ± 1.47	0.24 ± 4.80	5.82 ± 3.46
	T1	1.65 ± 1.65	1.08 ± 0.83	−1.93 ± 1.22	0.69 ± 1.17	−1.04 ± 0.95	3.92 ± 1.02
	*p*-value	0.070 ^c^	0.000 ^a^	0.000 ^b^	0.000 ^a^	0.509 ^c^	0.000 ^a^

**Table A19:** Transverse plane mean and standard deviation angles of different joints (hip, pelvis, knee and ankle), at T0 and T1, at the heel strike (HS), mid stance (MD), and toe off (TO), for both lower limbs, along with the *p*-value between both assessment periods. The effect size is presented as large (^a^), moderate (^b^) or small (^c^).

Joint Angle (Degrees)	Time of Assessment	Left Lower Limb			Right Lower Limb		
		HS	MD	TO	HS	MD	TO
Pelvis	T0	84.81 ± 2.62	87.49 ± 1.95	93.89 ± 2.17	93.89 ± 2.07	93.51 ± 2.01	86.01 ± 2.63
	T1	84.55 ± 3.14	85.41 ± 2.11	92.94 ± 3.45	92.11 ± 2.30	92.35 ± 2.71	84.03 ± 2.16
	*p*-value	0.000 ^a^	0.000 ^a^	0.294 ^c^	0.001 ^a^	0.062 ^c^	0.008 ^a^
Left Hip	T0	−14.13 ± 2.01	−12.67 ± 0.96	−8.90 ± 1.53	−10.84 ± 1.36	−10.26 ± 1.08	−6.94 ± 3.07
	T1	−13.00 ± 4.62	−7.60 ± 1.65	−3.13 ± 4.10	−5.82 ± 1.81	−0.08 ± 1.95	−4.76 ± 2.54
	*p*-value	0.017 ^a^	0.000 ^a^	0.000 ^a^	0.000 ^a^	0.000 ^a^	0.001 ^b^
Right Hip	T0	13.18 ± 4.99	8.07 ± 3.79	12.95 ± 4.49	8.91 ± 3.14	11.5 ± 4.79	14.3 ± 4.77
	T1	0.31 ± 4.74	−3.78 ± 3.32	−2.79 ± 2.51	−6.39 ± 5.65	−2.41 ± 2.22	−0.37 ± 3.74
	*p*-value	0.000 ^a^	0.000 ^a^	0.000 ^a^	0.000 ^a^	0.000 ^a^	0.000 ^a^
Left Knee	T0	−5.54 ± 2.45	2.64 ± 2.19	−7.47 ± 4.13	10.88 ± 2.25	−11.05 ± 1.74	−7.20 ± 2.02
	T1	−15.04 ± 3.10	−8.83 ± 1.86	−11.05 ± 2.27	−3.21 ± 1.83	−15.86 ± 2.11	−15.77 ± 2.36
	*p*-value	0.000 ^a^	0.000 ^a^	0.000 ^a^	0.000 ^a^	0.000 ^a^	0.000 ^a^
Right Knee	T0	4.22 ± 6.81	−25.63 ± 15.2	−32.39 ± 15.18	−30.58 ± 13.86	−27.93 ± 18.71	−30.00 ± 14.83
	T1	−14.94 ± 7.28	−15.91 ± 2.44	−20.92 ± 2.98	−19.71 ± 2.86	−17.64 ± 3.38	−17.25 ± 2.93
	*p*-value	0.000 ^c^	0.052 ^c^	0.000 ^c^	0.000 ^c^	0.001 ^c^	0.001 ^b^
Left Ankle	T0	1.47 ± 1.46	−1.54 ± 1.93	5.93 ± 3.94	−6.21 ± 2.30	6.62 ± 1.93	−0.73 ± 2.36
	T1	9.19 ± 1.70	1.09 ± 1.21	4.36 ± 2.70	1.05 ± 1.06	6.34 ± 1.48	0.73 ± 1.26
	*p*-value	0.000 ^a^	0.000 ^a^	0.038 ^a^	0.000 ^a^	0.000 ^c^	0.002 ^b^
Right Ankle	T0	−13.10 ± 23.26	−1.69 ± 16.01	−9.28 ± 17.48	−6.77 ± 15.44	−9.95 ± 21.76	−3.35 ± 17.26
	T1	5.48 ± 2.22	7.41 ± 1.63	3.17 ± 1.57	9.15 ± 3.21	3.08 ± 1.49	4.52 ± 1.42
	*p*-value	0.000 ^b^	0.000 ^b^	0.000 ^b^	0.000 ^b^	0.000 ^b^	0.000 ^b^

P3 shows a clear delayed toe-off for both limbs at T1. It exhibits a generally higher pelvic angle at T1, than T0, both in retroversion when compared with the reference data, but within the normal 5 degrees of range of motion. In the transverse plane it is possible to see that at T1 the rotation is minimal and T0 shows a 10-degree variation. This patient shows a prominent hip extension in comparison to the reference population, although hip flexion remains within reference values. The frontal range of motion is also within reference values. In the transverse plane hip rotation is smooth but more internal for the right lower limb cycles at T0. With regards to the knee angle, the stance phase knee flexion and the following extension are higher than the reference data. The swing phase knee flexion is slightly higher than the reference. The frontal variation of the knee rotation in swing phase is also higher than 10 degrees at T0. The transverse plane shows a tendency for the right lower limb cycles to show a more external rotation of the knee and the left lower limb cycles a more internal rotation. The ankle angle of P3 exhibits an almost permanent dorsiflexion, with a peak higher than the reference gait data. In the frontal plane, eversion/inversion transition is stricter and less regular at T1. The transverse plane, shows a generally more prominent internal rotation of the ankle than in the used reference population.

### Appendix C.4. Participant 4

**Table A20:** Sagittal plane mean and standard deviation angles of different joints (hip, pelvis, knee and ankle), at T0 and T1, at the heel strike (HS), mid stance (MD), and toe off (TO), for both lower limbs, along with the *p*-value between both assessment periods. The effect size is presented as large (^a^), moderate (^b^) or small (^c^).

Joint Angle (Degrees)	Time of Assessment	Left Lower Limb			Right Lower Limb		
		HS	MD	TO	HS	MD	TO
Pelvis	T0	−0.35 ± 1.15	0.36 ± 1.08	−0.06 ± 1.03	−1.55 ± 1.16	−3.60 ± 1.25	−4.80 ± 1.07
	T1	4.89 ± 1.24	3.63 ± 0.60	5.67 ± 0.86	3.25 ± 1.18	3.04 ± 0.87	−0.40 ± 1.16
	*p*-value	0.000 ^a^	0.001 ^a^	0.000 ^a^	0.000 ^a^	0.001 ^a^	0.000 ^a^
Left Hip	T0	30.09 ± 1.93	7.93 ± 2.89	0.38 ± 6.70	−9.56 ± 5.68	34.19 ± 2.67	23.78 ± 2.61
	T1	24.82 ± 2.39	0.02 ± 0.65	−9.10 ± 3.99	−17.18 ± 1.97	24.8 ± 2.20	16.15 ± 5.72
	*p*-value	0.000 ^a^	0.000 ^a^	0.000 ^a^	0.000 ^a^	0.000 ^a^	0.000 ^a^
Right Hip	T0	−3.29 ± 3.36	32.03 ± 2.13	27.15 ± 2.28	31.33 ± 1.45	14.25 ± 1.65	0.44 ± 3.10
	T1	−15.27 ± 2.92	23.91 ± 1.26	20.71 ± 2.05	25.20 ± 1.97	5.53 ± 1.48	−9.06 ± 6.02
	*p*-value	0.000 ^a^	0.000 ^a^	0.000 ^a^	0.000 ^a^	0.000 ^a^	0.000 ^a^
Left Knee	T0	20.48 ± 3.48	18.46 ± 3.54	41.9 ± 5.96	12.61 ± 5.05	65.36 ± 4.20	29.78 ± 2.65
	T1	19.08 ± 3.06	15.91 ± 1.81	38.49 ± 4.20	8.59 ± 3.91	62.45 ± 2.89	28.74 ± 4.86
	*p*-value	0.024 ^c^	0.014 ^b^	0.005 ^b^	0.000 ^b^	0.094 ^a^	0.288 ^c^
Right Knee	T0	−4.05 ± 2.11	70.39 ± 4.08	35.19 ± 1.94	25.25 ± 1.99	27.48 ± 2.00	45.84 ± 3.07
	T1	5.51 ± 4.50	23.91 ± 1.26	30.65 ± 3.07	18.87 ± 4.57	21.05 ± 1.92	40.81 ± 6.26
	*p*-value	0.000 ^a^	0.000 ^a^	0.000 ^a^	0.000 ^a^	0.001 ^b^	0.000 ^a^
Left Ankle	T0	3.62 ± 3.58	11.63 ± 3.34	−0.72 ± 2.24	18.67 ± 3.70	−2.96 ± 3.82	6.61 ± 3.54
	T1	−0.77 ± 1.63	9.64 ± 0.86	2.31 ± 3.30	15.85 ± 1.50	−4.55 ± 2.25	6.33 ± 2.05
	*p*-value	0.800 ^c^	0.116 ^b^	0.000 ^b^	0.312 ^c^	0.331 ^c^	0.372 ^c^
Right Ankle	T0	20.1 ± 2.19	−4.37 ± 2.03	6.23 ± 1.55	−4.74 ± 1.37	12.64 ± 1.37	10.55 ± 2.28
	T1	18.01 ± 2.70	−2.58 ± 2.02	5.97 ± 2.08	−3.27 ± 1.65	12.16 ± 1.72	5.07 ± 3.72
	*p*-value	0.001 ^b^	0.222 ^a^	0.545 ^c^	0.000 ^b^	0.354 ^b^	0.000 ^a^

**Table A21:** Frontal plane mean and standard deviation angles of different joints (hip, pelvis, knee and ankle), at T0 and T1, at the heel strike (HS), mid stance (MD), and toe off (TO), for both lower limbs, along with the *p*-value between both assessment periods. The effect size is presented as large (^a^), moderate (^b^) or small (^c^).

Joint Angle (Degrees)	Time of Assessment	Left Lower Limb			Right Lower Limb		
		HS	MD	TO	HS	MD	TO
Pelvis	T0	−0.69 ± 1.66	−3.20 ± 1.95	−2.72 ± 2.4	−3.58 ± 1.86	−1.36 ± 1.84	−2.99 ± 1.88
	T1	−2.89 ± 1.61	−5.32 ± 0.57	−4.70 ± 2.3	−4.58 ± 1.87	−3.72 ± 1.48	−5.44 ± 1.98
	*p*-value	0.000 ^a^	0.037 ^a^	0.000 ^b^	0.002 ^b^	0.024 ^a^	0.000 ^a^
Left Hip	T0	3.49 ± 3.20	11.28 ± 2.51	5.41 ± 2.16	12.79 ± 2.31	4.36 ± 1.99	12.00 ± 2.12
	T1	0.48 ± 2.40	7.31 ± 1.37	1.29 ± 1.65	7.25 ± 1.86	1.61 ± 1.06	9.26 ± 1.50
	*p*-value	0.000 ^b^	0.028 ^a^	0.000 ^a^	0.000 ^a^	0.002 ^a^	0.000 ^b^
Right Hip	T0	4.87 ± 1.71	−4.88 ± 1.54	2.27 ± 1.22	−2.54 ± 1.96	2.17 ± 1.29	−7.83 ± 1.53
	T1	10.39 ± 1.93	−0.45 ± 0.59	9.90 ± 1.58	3.52 ± 1.70	9.51 ± 1.63	−1.15 ± 1.36
	*p*-value	0.000 ^b^	0.028 ^a^	0.000 ^a^	0.000 ^a^	0.000 ^a^	0.005 ^b^
Left Knee	T0	−6.98 ± 6.74	−12.45 ± 4.17	−11.81 ± 10.89	−12.72 ± 8.29	−1.44 ± 9.61	−3.29 ± 6.73
	T1	−9.4 ± 1.05	−9.27 ± 0.35	−0.43 ± 2.30	−11.13 ± 0.81	3.92 ± 1.39	−5.09 ± 2.24
	*p*-value	0.018 ^c^	0.000 ^a^	0.000 ^b^	0.000 ^b^	0.000 ^a^	0.196 ^c^
Right Knee	T0	−10.36 ± 4.95	3.73 ± 4.66	0.16 ± 2.71	−0.91 ± 2.64	−3.57 ± 2.57	−5.49 ± 3.45
	T1	−10.24 ± 0.81	−0.45 ± 0.59	−6.58 ± 1.24	−7.22 ± 1.53	−7.28 ± 0.64	−5.77 ± 1.54
	*p*-value	0.000 ^a^	0.000 ^a^	0.000 ^b^	0.000 ^b^	0.000 ^a^	0.000 ^c^
Left Ankle	T0	6.90 ± 6.84	3.08 ± 7.54	−1.86 ± 1.61	6.41 ± 6.41	4.03 ± 7.37	−0.27 ± 7.07
	T1	6.93 ± 1.78	2.99 ± 1.38	7.40 ± 2.95	5.28 ± 1.69	6.98 ± 1.29	1.02 ± 1.35
	*p*-value	0.186 ^c^	0.028 ^c^	0.000 ^a^	0.918 ^c^	0.001 ^b^	0.000 ^b^
Right Ankle	T0	6.49 ± 1.58	−3.70 ± 1.61	0.73 ± 1.45	3.55 ± 1.58	1.02 ± 1.46	2.22 ± 1.83
	T1	4.71 ± 1.28	−0.19 ± 1.80	0.39 ± 1.43	1.49 ± 2.01	0.83 ± 1.42	2.38 ± 2.23
	*p*-value	0.000 ^a^	0.054 ^a^	0.226 ^c^	0.000 ^a^	0.063 ^a^	0.707 ^c^

**Table A22:** Transverse plane mean and standard deviation angles of different joints (hip, pelvis, knee and ankle), at T0 and T1, at the heel strike (HS), mid stance (MD), and toe off (TO), for both lower limbs, along with the *p*-value between both assessment periods. The effect size is presented as large (^a^), moderate (^b^) or small (^c^).

Joint Angle (Degrees)	Time of Assessment	Left Lower Limb			Right Lower Limb		
		HS	MD	TO	HS	MD	TO
Pelvis	T0	84.10 ± 2.74	86.55 ± 2.16	92.58 ± 2.47	92.06 ± 2.52	91.16 ± 2.52	84.67 ± 2.37
	T1	88.67 ± 2.29	91.79 ± 1.51	93.44 ± 2.43	95.43 ± 2.33	94.31 ± 2.16	89.17 ± 2.43
	*p*-value	0.000 ^a^	0.017 ^a^	0.121 ^c^	0.000 ^a^	0.001 ^a^	0.000 ^a^
Left Hip	T0	−7.3 ± 9.13	−13.23 ± 6.89	−15.61 ± 11.6	−12.11 ± 18.20	−10.99 ± 10.06	−3.00 ± 9.21
	T1	4.65 ± 3.86	3.21 ± 1.01	5.06 ± 2.58	5.76 ± 2.98	4.38 ± 1.37	5.14 ± 2.33
	*p*-value	0.000 ^a^	0.028 ^a^	0.000 ^a^	0.000 ^a^	0.001 ^a^	0.000 ^b^
Right Hip	T0	−6.31 ± 3.82	−6.06 ± 4.59	−1.60 ± 3.43	0.05 ± 3.95	−3.72 ± 3.89	−10.28 ± 3.96
	T1	−6.25 ± 2.87	−10.03 ± 2.17	−8.66 ± 2.58	−12.33 ± 5.91	−6.23 ± 3.23	−5.32 ± 3.52
	*p*-value	0.725 ^c^	0.249 ^b^	0.000 ^a^	0.000 ^a^	0.683 ^c^	0.000 ^a^
Left Knee	T0	−13.98 ± 9.31	−4.46 ± 5.52	−10.22 ± 7.02	0.37 ± 24.10	−18.15 ± 5.28	−16.34 ± 8.27
	T1	−19.26 ± 2.16	−16.13 ± 2.64	−31.03 ± 2.76	−16.47 ± 1.89	−25.14 ± 3.96	−18.35 ± 2.36
	*p*-value	0.001 ^a^	0.096 ^c^	0.000 ^a^	0.000 ^a^	0.003 ^a^	0.036 ^c^
Right Knee	T0	−3.29 ± 3.36	−8.49 ± 3.60	−13.66 ± 3.53	−18.66 ± 4.47	−10.95 ± 4.33	−4.46 ± 4.38
	T1	6.10 ± 2.03	−10.03 ± 2.17	0.40 ± 1.93	−0.99 ± 1.93	2.06 ± 1.49	−1.52 ± 1.55
	*p*-value	0.000 ^a^	0.052 ^a^	0.000 ^a^	0.000 ^a^	0.001 ^a^	0.001 ^b^
Left Ankle	T0	4.29 ± 4.25	−29.49 ± 2.14	−0.02 ± 1.87	−29.40 ± 3.66	−27.30 ± 3.78	−30.90 ± 2.87
	T1	−24.37 ± 0.83	−28.99 ± 0.30	−30.84 ± 1.39	−28.51 ± 1.11	−24.47 ± 0.92	−30.25 ± 1.04
	*p*-value	0.399 ^c^	0.046 ^a^	0.000 ^b^	0.000 ^c^	0.000 ^c^	0.041 ^c^
Right Ankle	T0	−6.65 ± 2.35	−9.04 ± 2.47	−9.11 ± 3.16	−1.70 ± 2.88	−8.20 ± 2.41	−7.32 ± 2.66
	T1	−18.05 ± 1.05	−10.21 ± 0.68	−19.78 ± 1.81	−13.49 ± 1.57	−19.39 ± 1.32	−17.46 ± 2.79
	*p*-value	0.000 ^a^	0.506 ^b^	0.000 ^a^	0.000 ^a^	0.001 ^a^	0.000 ^a^

P4 shows similar toe-off moments at T0 and T1. The pelvic angle shows a slight retroversion, when compared to the reference data, and a generally lower retroversion at T1, than T0. In the frontal plane, obliquity is within the normal 5° variation, and in the transverse plane left and right rotations are more prominent at T1 than T0 and the variation is around −5 to 5 degrees which is near the reference pattern. P4′s hip extension, is more prominent than the reference population. The frontal range of motion is within reference values and, in the transverse plane, an excessive rotation (more than 10 degrees total displacement) is observed at T0. T1, on the contrary, exhibits slightly more external but smoother rotations, than the reference gait data, to sustain body weight. The stance phase knee flexion is higher than the reference population, and the following extension is lower. Swing phase knee flexion is also higher than the reference. In the transverse plane it is possible to see an asymmetry at T1 with the right lower limb cycles presenting a more internal knee rotation and the left lower limb cycles a more external knee rotation throughout the whole gait cycle. The ankle angle shows a higher dorsiflexion (up to 25°) than the reference data. In the frontal plane eversion is less strict than the other patients and within the reference values. The transverse plane shows almost no alterations on ankle rotation during the gait cycle for each assessment, despite right lower limb cycles being more within the normal rotation range and the left lower limb cycles more externally rotated.

### Appendix C.5. Participant 5

**Table A23:** Sagittal plane mean and standard deviation angles of different joints (hip, pelvis, knee and ankle), at T0 and T1, at the heel strike (HS), mid stance (MD), and toe off (TO), for both lower limbs, along with the *p*-value between both assessment periods. The effect size is presented as large (^a^), moderate (^b^) or small (^c^).

Joint Angle (Degrees)	Time of Assessment	Left Lower Limb			Right Lower Limb		
		HS	MD	LTO	HS	MD	TO
Pelvis	T0	−1.73 ± 1.63	−1.81 ± 1.10	5.74 ± 22.57	−3.85 ± 2.80	−2.01 ± 0.69	−4.50 ± 1.08
	T1	1.58 ± 0.79	1.10 ± 0.88	5.30 ± 0.91	1.03 ± 1.41	0.19 ± 0.94	−2.14 ± 0.82
	*p*-value	0.000 ^a^	0.000 ^a^	0.000 ^a^	0.000 ^a^	0.000 ^a^	0.000 ^a^
Left Hip	T0	21.24 ± 1.69	−4.48 ± 1.33	−7.81 ± 24.89	−19.64 ± 2.11	23.59 ± 1.53	17.83 ± 1.39
	T1	27.16 ± 2.50	1.66 ± 1.93	−4.68 ± 3.14	−13.89 ± 1.74	29.02 ± 1.57	22.93 ± 4.11
	*p*-value	0.000 ^a^	0.000 ^a^	0.000 ^a^	0.000 ^a^	0.000 ^a^	0.000 ^a^
Right Hip	T0	−20.86 ± 1.49	17.43 ± 1.40	23.55 ± 19.51	22.63 ± 1.63	−4.68 ± 1.70	−17.47 ± 1.40
	T1	−15.33 ± 2.55	24.06 ± 1.45	26.08 ± 3.16	30.13 ± 2.93	2.31 ± 2.74	−10.57 ± 4.49
	*p*-value	0.000 ^a^	0.000 ^a^	0.000 ^a^	0.000 ^a^	0.000 ^a^	0.000 ^a^
Left Knee	T0	13.69 ± 2.13	12.97 ± 2.01	39.18 ± 2.21	12.96 ± 1.74	66.3 ± 1.79	24.01 ± 2.15
	T1	14.71 ± 4.16	16.05 ± 4.04	47.93 ± 5.89	15.14 ± 3.6	29.02 ± 1.57	30.21 ± 3.78
	*p*-value	0.257 ^c^	0.001 ^b^	0.000 ^a^	0.006 ^b^	0.000 ^a^	0.000 ^a^
Right Knee	T0	4.87 ± 1.71	59.10 ± 2.13	26.25 ± 2.79	16.51 ± 1.85	11.39 ± 2.58	33.90 ± 2.19
	T1	11.93 ± 1.89	60.38 ± 2.31	29.79 ± 2.83	17.95 ± 3.05	13.87 ± 2.49	39.85 ± 5.87
	*p*-value	0.013 ^b^	0.015 ^a^	0.000 ^a^	0.004 ^a^	0.002 ^a^	0.000 ^a^
Left Ankle	T0	1.11 ± 1.10	9.15 ± 1.20	7.23 ± 2.81	25.02 ± 1.14	−6.15 ± 1.07	−1.54 ± 2.09
	T1	−3.11 ± 0.81	12.06 ± 1.78	2.45 ± 4.81	27.33 ± 1.98	−2.65 ± 1.33	4.15 ± 2.28
	*p*-value	0.000 ^a^	0.000 ^a^	0.000 ^a^	0.000 ^a^	0.000 ^a^	0.000 ^a^
Right Ankle	T0	20.25 ± 1.30	2.21 ± 0.81	2.98 ± 1.55	−0.01 ± 1.02	10.82 ± 1.22	6.89 ± 2.27
	T1	21.24 ± 1.34	−1.37 ± 1.33	4.44 ± 1.94	−2.68 ± 0.85	11.05 ± 1.28	3.53 ± 3.93
	*p*-value	0.005 ^a^	0.000 ^a^	0.002 ^a^	0.000 ^a^	0.371 ^c^	0.000 ^a^

**Table A24:** Frontal plane mean and standard deviation angles of different joints (hip, pelvis, knee and ankle), at T0 and T1, at the heel strike (HS), mid stance (MD), and toe off (TO), for both lower limbs, along with the *p*-value between both assessment periods. The effect size is presented as large (^a^), moderate (^b^) or small (^c^).

Joint Angle (Degrees)	Time of Assessment	Left Lower Limb			Right Lower Limb		
		HS	MD	TO	HS	MD	TO
Pelvis	T0	−6.20 ± 1.40	−8.65 ± 1.68	−6.35 ± 1.25	−5.75 ± 1.27	−7.31 ± 1.09	−8.14 ± 1.43
	T1	−1.15 ± 1.07	−3.34 ± 1.92	−1.67 ± 3.12	−0.68 ± 2.47	−1.87 ± 2.22	−4.33 ± 1.33
	*p*-value	0.000 ^a^	0.000 ^a^	0.000 ^a^	0.000 ^a^	0.000 ^a^	0.000 ^a^
Left Hip	T0	1.58 ± 1.93	5.28 ± 1.13	−3.48 ± 1.31	3.87 ± 2.51	2.09 ± 0.81	5.56 ± 1.84
	T1	−3.01 ± 1.44	2.41 ± 1.62	−6.22 ± 1.67	0.50 ± 2.59	−1.10 ± 1.03	1.71 ± 1.84
	*p*-value	0.000 ^a^	0.000 ^a^	0.000 ^a^	0.000 ^a^	0.000 ^a^	0.434 ^b^
Right Hip	T0	4.11 ± 2.17	0.14 ± 1.16	3.56 ± 4.56	−3.23 ± 3.13	5.46 ± 0.88	−0.86 ± 1.50
	T1	5.42 ± 1.64	1.87 ± 1.78	6.39 ± 2.13	−0.51 ± 2.72	5.46 ± 2.14	−1.83 ± 1.53
	*p*-value	0.001 ^b^	0.001 ^b^	0.000 ^b^	0.001 ^b^	0.829 ^c^	0.000 ^b^
Left Knee	T0	−0.89 ± 0.57	−0.97 ± 0.46	−7.42 ± 1.45	−1.56 ± 0.52	−4.24 ± 1.51	−1.26 ± 0.96
	T1	−0.59 ± 0.67	−0.27 ± 0.61	−2.45 ± 4.06	−0.96 ± 0.66	−1.10 ± 1.03	0.91 ± 1.26
	*p*-value	0.097 ^b^	0.000 ^b^	0.000 ^b^	0.000 ^a^	0.000 ^a^	0.000 ^a^
Right Knee	T0	−6.31 ± 3.82	7.91 ± 1.79	3.23 ± 2.14	0.86 ± 0.58	−0.32 ± 0.37	0.67 ± 0.66
	T1	−2.17 ± 0.64	2.31 ± 1.01	−0.70 ± 1.16	−1.48 ± 0.86	−1.48 ± 0.69	−1.18 ± 1.40
	*p*-value	0.000 ^a^	0.000 ^a^	0.000 ^a^	0.000 ^a^	0.000 ^a^	0.000 ^a^
Left Ankle	T0	0.97 ± 0.96	−4.53 ± 1.00	−4.10 ± 1.35	−5.00 ± 0.91	−5.30 ± 1.06	−3.57 ± 1.24
	T1	2.74 ± 1.00	0.06 ± 1.23	0.46 ± 1.11	0.50 ± 1.49	−2.62 ± 0.80	−1.26 ± 1.16
	*p*-value	0.000 ^a^	0.000 ^a^	0.000 ^a^	0.000 ^a^	0.000 ^a^	0.000 ^a^
Right Ankle	T0	3.76 ± 1.11	−4.05 ± 0.53	−2.72 ± 1.27	1.71 ± 0.78	−1.47 ± 1.18	3.40 ± 1.58
	T1	0.90 ± 1.32	−3.73 ± 2.05	−2.71 ± 1.84	2.84 ± 1.70	−1.76 ± 1.20	1.49 ± 2.14
	*p*-value	0.000 ^a^	0.014 ^b^	0.857 ^c^	0.001 ^a^	0.475 ^c^	0.005 ^a^

**Table A25:** Transverse plane mean and standard deviation angles of different joints (hip, pelvis, knee and ankle), at T0 and T1, at the heel strike (HS), mid stance (MD), and toe off (TO), for both lower limbs, along with the *p*-value between both assessment periods. The effect size is presented as large (^a^) or moderate (^b^).

Joint Angle (Degrees)	Time of Assessment	Left Lower Limb			Right Lower Limb		
		HS	MD	TO	HS	MD	TO
Pelvis	T0	89.55 ± 1.64	92.86 ± 1.72	101.39 ± 8.13	99.45 ± 1.62	94.12 ± 1.34	89.76 ± 1.72
	T1	87.40 ± 1.72	90.58 ± 1.68	97.02 ± 1.50	96.73 ± 1.84	92.25 ± 1.52	87.21 ± 1.90
	*p*-value	0.000 ^b^	0.000 ^a^	0.000 ^a^	0.000 ^a^	0.000 ^a^	0.000 ^a^
Left Hip	T0	−18.62 ± 3.40	−11.72 ± 1.90	−8.22 ± 13.92	−9.09 ± 1.78	−4.08 ± 2.11	−14.53 ± 2.78
	T1	−12.83 ± 2.52	−5.93 ± 3.84	−1.01 ± 4.84	−4.63 ± 3.56	6.23 ± 3.15	−9.73 ± 2.15
	*p*-value	0.000 ^a^	0.000 ^a^	0.000 ^a^	0.000 ^a^	0.000 ^a^	0.000 ^a^
Right Hip	T0	−0.65 ± 1.89	4.60 ± 2.77	−5.24 ± 15.06	−5.13 ± 2.38	−0.43 ± 1.97	−1.67 ± 1.75
	T1	−3.22 ± 2.36	−0.55 ± 2.91	−10.39 ± 2.25	−13.44 ± 2.00	−4.66 ± 2.86	−4.18 ± 2.01
	*p*-value	0.000 ^a^	0.000 ^a^	0.000 ^a^	0.000 ^a^	0.000 ^a^	0.000 ^a^
Left Knee	T0	8.93 ± 2.54	13.96 ± 1.13	17.25 ± 1.62	18.06 ± 1.45	13.81 ± 1.43	10.67 ± 2.37
	T1	−8.55 ± 2.36	−4.18 ± 2.24	−0.76 ± 2.50	−0.60 ± 1.31	6.23 ± 3.15	−5.94 ± 1.92
	*p*-value	0.000 ^a^	0.000 ^a^	0.000 ^a^	0.000 ^a^	0.000 ^a^	0.000 ^a^
Right Knee	T0	13.57 ± 2.83	−18.78 ± 1.95	−25.08 ± 3.46	−27.53 ± 1.90	−21.18 ± 1.41	−20.37 ± 1.50
	T1	−2.96 ± 1.42	−6.50 ± 2.28	−7.25 ± 2.93	−11.38 ± 3.11	−6.39 ± 2.17	−7.66 ± 2.59
	*p*-value	0.000 ^a^	0.000 ^a^	0.000 ^a^	0.000 ^a^	0.000 ^a^	0.000 ^a^
Left Ankle	T0	1.32 ± 1.30	−8.29 ± 1.80	−7.80 ± 2.33	−6.99 ± 1.65	−11.44 ± 1.12	−8.59 ± 2.03
	T1	10.12 ± 2.47	3.32 ± 2.68	3.44 ± 2.14	2.33 ± 1.55	2.02 ± 1.24	4.19 ± 2.29
	*p*-value	0.000 ^a^	0.000 ^a^	0.000 ^a^	0.000 ^a^	0.000 ^a^	0.000 ^a^
Right Ankle	T0	−1.42 ± 1.27	−3.86 ± 1.59	−1.92 ± 1.64	4.41 ± 0.87	−1.89 ± 1.46	1.09 ± 2.37
	T1	−15.64 ± 1.20	−15.86 ± 1.94	−16.46 ± 2.54	−6.61 ± 1.57	−15.76 ± 1.43	−12.62 ± 2.30
	*p*-value	0.000 ^a^	0.000 ^a^	0.000 ^a^	0.000 ^a^	0.000 ^a^	0.000 ^a^

P5 has a delayed toe-off at T1 and a peak pelvic anteversion during loading response at T0. This peak disappeared at T1 which showed a more retroverted pelvic angle in general. However, this angle is generally within the reference values (at T0) and has a 5 degrees variation, in the sagittal plane. In the transverse plane, right rotations are more prominent than those in the reference gait data, and left rotation is within reference values. Variation is around −5 to 10 degrees. Hip extension is more prominent than the used reference population, although hip flexion is lower than reference values. Excessive transverse rotation (more than 10 degrees total displacement) is also observed in this patient. The stance phase knee flexion is higher at T1 than T0 and also than the reference population, and the following extension is lower. Swing phase knee flexion is also higher than reference data and higher at T1 than T0. The transverse plane analysis shows a very smooth rotation and more external than the used reference gait data in the case of the right lower limb cycles at T0 as well as a more internal rotation in the case of left lower limb cycles, also at T0. The ankle angle shows a higher plantar-flexion than the reference population on the swing phase, and a higher dorsiflexion (up to 30°) before the toe-off. In the frontal plane, the eversion is more prominent than the correspondent for the reference values, and the swing phase were different for all the assessments.
